# Digestive exophagy of biofilms by intestinal amoeba and its impact on stress tolerance and cytotoxicity

**DOI:** 10.1038/s41522-023-00444-x

**Published:** 2023-10-09

**Authors:** Eva Zanditenas, Meirav Trebicz-Geffen, Divya Kolli, Laura Domínguez-García, Einan Farhi, Liat Linde, Diego Romero, Matthew Chapman, Ilana Kolodkin-Gal, Serge Ankri

**Affiliations:** 1 Department of Molecular Microbiology, Ruth and Bruce Rappaport Faculty of Medicine, Technion, Haifa, Israel; 2 Department of Molecular, Cellular, and Developmental Biology, University of Michigan, Ann Arbor, USA; 3 Departamento de Microbiología, Instituto de Hortofruticultura Subtropical y Mediterránea ‘La Mayora’, Universidad de Málaga-Consejo Superior de Investigaciones Científicas (IHSM-UMA-CSIC), Universidad de Málaga, Málaga, Spain; 4 Technion Genomics Center, Technion - Israel Institute of Technology, Haifa, Israel; 5 Department of Plant Pathology and Microbiology, the Robert H. Smith Faculty of Agriculture, Food & Environment, The Hebrew University of Jerusalem, Rehovot, Israel; 6 Scojen Institute for Synthetic Biology, Reichman University, Herzliya, Israel

**Keywords:** Biofilms, Environmental microbiology

## Abstract

The human protozoan parasite *Entamoeba histolytica* is responsible for amebiasis, a disease endemic to developing countries. *E. histolytica* trophozoites colonize the large intestine, primarily feeding on bacteria. However, in the gastrointestinal tract, bacterial cells form aggregates or structured communities called biofilms too large for phagocytosis. Remarkably, trophozoites are still able to invade and degrade established biofilms, utilizing a mechanism that mimics digestive exophagy. Digestive exophagy refers to the secretion of digestive enzymes that promote the digestion of objects too large for direct phagocytosis by phagocytes. *E. histolytica* cysteine proteinases (CPs) play a crucial role in the degradation process of *Bacillus subtilis* biofilm. These proteinases target TasA, a major component of the *B. subtilis* biofilm matrix, also contributing to the adhesion of the parasite to the biofilm. In addition, they are also involved in the degradation of biofilms formed by Gram-negative and Gram-positive enteric pathogens. Furthermore, biofilms also play an important role in protecting trophozoites against oxidative stress. This specific mechanism suggests that the amoeba has adapted to prey on biofilms, potentially serving as an untapped reservoir for novel therapeutic approaches to treat biofilms. Consistently, products derived from the amoeba have been shown to restore antibiotic sensitivity to biofilm cells. In addition, our findings reveal that probiotic biofilms can act as a protective shield for mammalian cells, hindering the progression of the parasite towards them.

## Introduction

*Entamoeba histolytica* is a protozoan parasite that causes amebiasis, a highly prevalent intestinal disease primarily found in developing countries. The transmission of amebiasis occurs through ingestion of contaminated food or water containing *E. histolytica* cysts, one of the two forms of the parasite^1^ *E. histolytica* cysts, one of the two forms of the parasite^1^. Upon entering the host’s intestine, the cyst, which is the parasite’s resilient form, undergoes excystation, releasing trophozoites, the active form of the parasite. In most cases, these trophozoites feed on the intestinal bacterial microbiota or cellular debris without causing symptoms. However, in symptomatic infections characterized by bloody diarrhea, the parasite disrupts the protective mucus layer and damages the epithelial cells of the intestine, triggering an inflammatory response. This response involves the recruitment of neutrophils and macrophages, which release reactive oxygen species and nitric oxide as part of the immune defense. Currently, there is no available vaccine for amebiasis, and the primary treatment option is metronidazole. However, metronidazole may have associated side effects such as diarrhea and anorexia^2,3^. Moreover, the emergence of metronidazole-resistant strains of *E. histolytica* raises concerns about the effectiveness of this treatment in the field^4^. The human large intestine is home to an estimated 10^14^ microorganisms, and many studies have highlighted the significant role of gut bacteria in the development of amebiasis (for a recent review, see ref. ^5^). These studies have examined the interaction of planktonic bacteria with *E. histolytica* trophozoites; yet, the interaction of this parasite with bacterial biofilms remained poorly characterized. This uncharted area is of high ecological and clinical relevance as in the intestinal tract, bacteria reside as complex microbial communities that are not planktonic. Instead, microbiota members form higher order structures named biofilms. The bacteria are embedded in complex, self-produced extracellular matrix (ECM) composed of polymeric substances (EPS), such as proteins, polysaccharides, extracellular DNA (eDNA), and lipids^6^ who are responsible for the adherence to each other and to biotic or abiotic surfaces^7,8^. Biofilms can arise from single bacterial species or from various bacterial species including both Gram-positive and Gram-negative bacteria^9^. Biofilms have been recognized to play a role in several conditions affecting the gut, including colorectal cancer, gut wounds, and inflammatory bowel diseases where *Bacteroides fragilis*-based biofilms dominated^10^. The presence of biofilms in the healthy gut was a subject of debate until recently. Recent advancements in preserving glycocalyx structures within these biofilms during the biological samples fixation process have enabled direct observation, providing concrete evidence of biofilms’ presence within the normal gut^11,12^. Notably, biofilms on mucosal surfaces in the colon tissues of healthy individuals display unique compositions, with Bacteroidetes, Lachnospiraceae, and Enterobacteriaceae predominantly inhabiting the right ascending colon, while Bacteroidetes and Lachnospiraceae prevail in the left descending colon^13^.

*Bacillus subtilis*, a Gram-positive bacterium, living in the rhizosphere, is a proficient biofilm former^14^. *B. subtilis* is also found in the human gastrointestinal tract, as it is wildly used in traditional fermented foods of many east Asian cultures for centuries^15^ and is an emerging probiotic to promote digestive health and a healthy immune system^16–18^. Within biofilms, the most abundant components of the exopolymers are carbohydrate-rich polymers (i.e., extracellular polysaccharides or exopolysaccharides), and proteins^19^. Within the biofilm of *B. subtilis*, TasA and TapA serve as the proteinaceous components of the extracellular matrix, playing an indispensable role in providing the biofilm with both rigidity and its complicated 3D architecture^19^. TasA forms β-sheet rich fibrils^20–22^ that are attached to the cell wall and, in conjunction with other extracellular components, promote cell-cell adhesion^21,23^. Biofilm formation and matrix production in *B. subtilis* was shown to protect it from various stressors, including antibiotics^24^, sodium hypochlorite, and ethanol^23,25^. Within biofilms formed by *Escherichia coli*^26^ and *Salmonella enterica* serotype Typhimurium^27^, curli protein fibers mediate cell-cell adhesion. Protein adhesins also hold together biofilms of enteric pathogens such as *Enterococcus faecalis*, held together by enterococcal surface protein (Esp), capable of forming fibers^28^ as well as Biofilm-Associated Proteins (Bap proteins)^6^. The importance of these adhesins is manifested by the protease sensitivity of *E. faecalis* biofilms^29^.

To provide mechanistic insights into protist predator interactions with biofilms, we characterized the molecular and physiological interactions between *E. histolytica* and the biofilm prey of *B. subtilis*. Our results indicate that biofilm-protist interactions are fundamentally different from the interactions between the parasites and planktonic cells. The role of CPs in the degradation of biofilms was well conserved and could be demonstrated for biofilms of the enteric pathogens *E. coli, S. Typhimurium*, and *E. faecalis*. Furthermore, these predator-prey interactions are unexpected regulators of stress tolerance and persistence for both the parasite predator and the prey bacteria.

## Results

### *E. histolytica* degrades *B. subtilis* biofilms in a dose and time-dependent manner

**Fig. 1:**
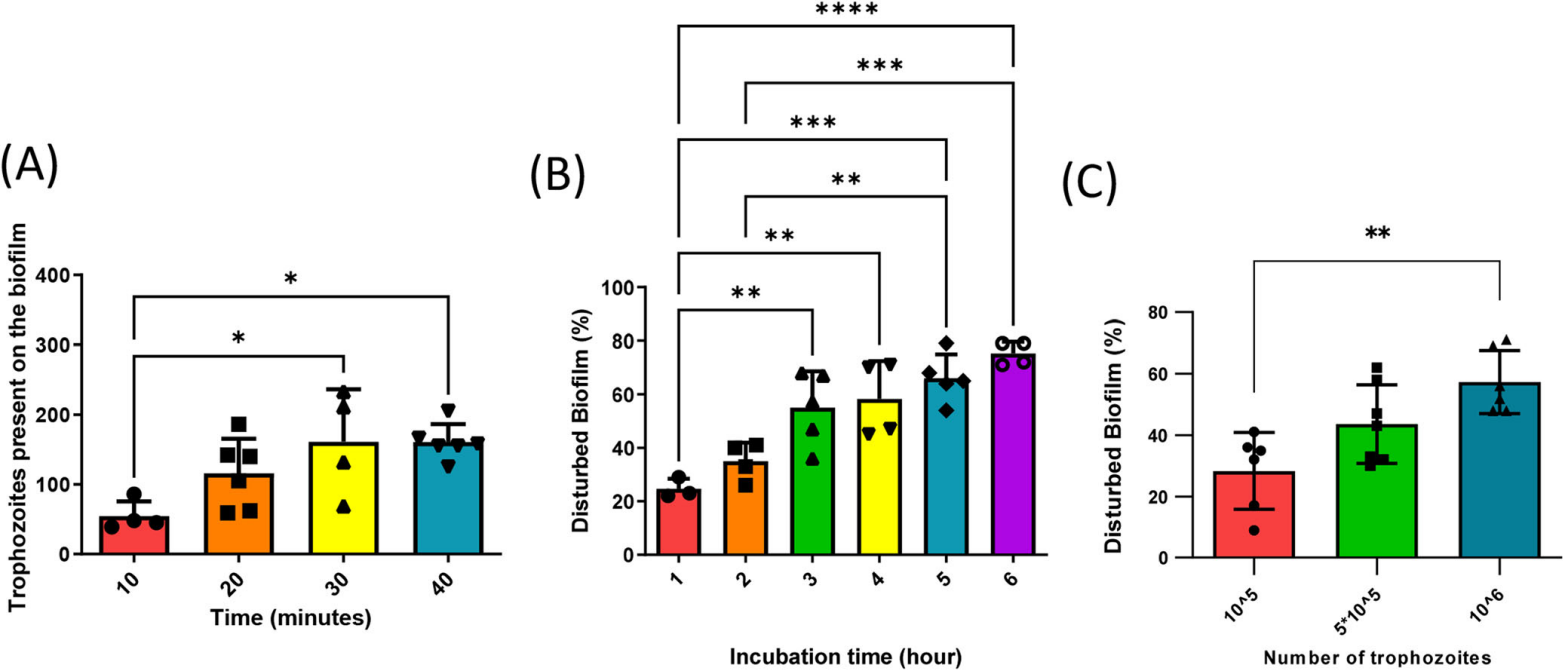
Digestive exophagy of *B. subtilis* biofilms by *E. histolytica*. **A** The number of trophozoites attached to *B. subtili*s biofilm was determined after 10, 20, 30, and 40 min of incubation at 37 °C as described in the “Methods” section. One-way ANOVA test was performed, *p < 0.05. ***p < 0.001). Data represent averages of results from three biological replicates. **B**, **C** Time and dose-dependent degradation of *B. subtilis* biofilms by *E. histolytica* trophozoites. GFP intensity of each biofilm was measured using ImageJ. One-way ANOVA test was performed, *p < 0.05, **p < 0.01, and ***p < 0.001. Data represent the average results from three biological replicates.

**Fig. 2:**
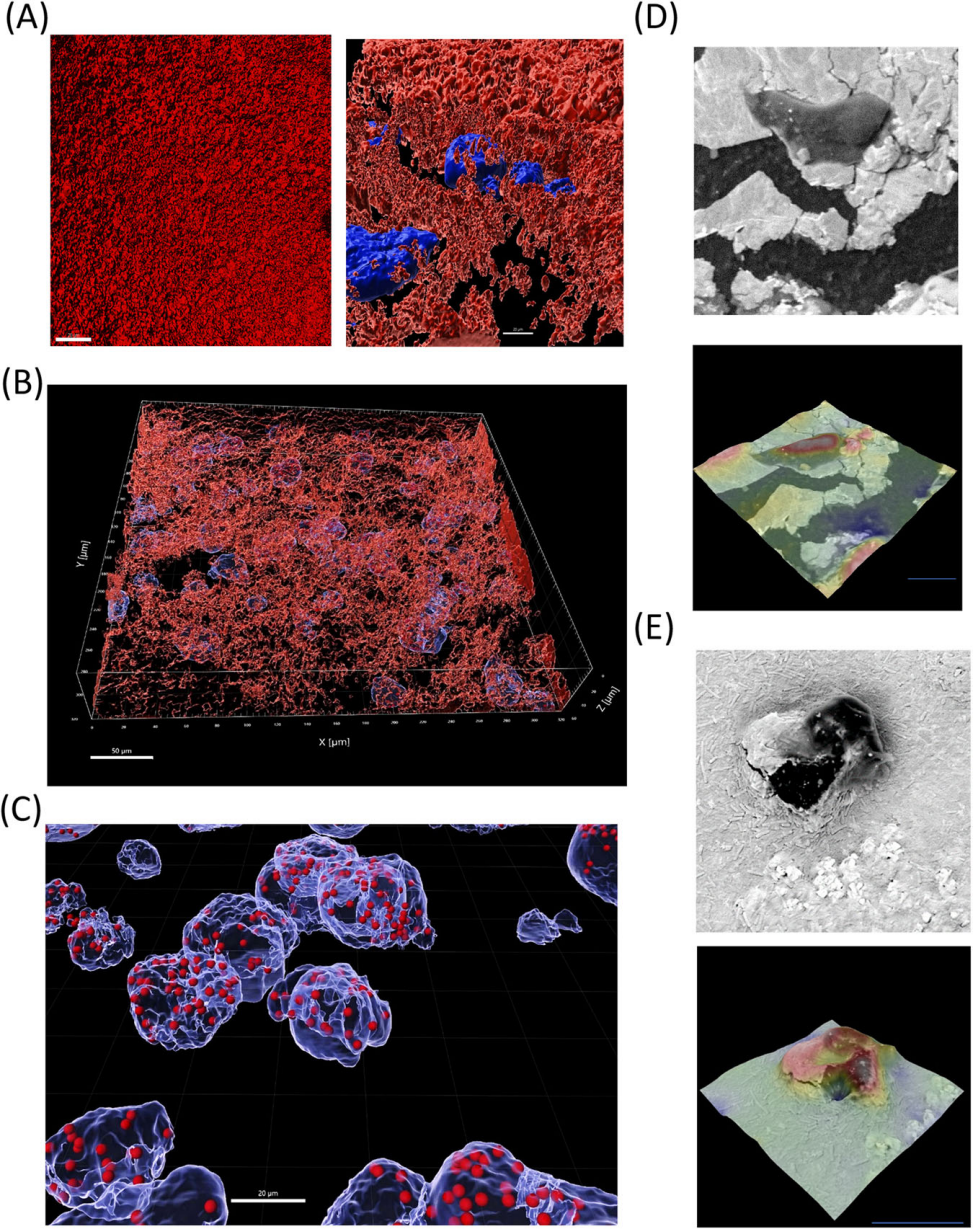
Biofilm degradation by *E. histolytica* is an active process. **A** Confocal microscopy (X30) of *B. subtilis* NCIB3610 biofilm cells carrying TasA-mCherry after 180 min of incubation at 37 °C, incubated with or without *E. histolytica* trophozoites (stained with DAPI). **B** 3D imaging of *B. subtilis* biofilm (expressing TasA-mCherry) containing trophozoites (stained with DAPI) from confocal microscopy images. After analyzing with Imaris software, bacteria and pieces of biofilm ingested by the trophozoites could be detected inside trophozoites and were visible as red dots. **C** Zoom on trophozoites (in blue) having ingested bacteria from the biofilm (shown as red dots). **D** Electron microscopy images of trophozoites present on the biofilm. Upper panel: Trophozoites (in dark grey) are observed on the surface of the biofilm (in grey). Lower panel: Heat map of the upper panel by height- red (high) to blue (low), illustrating trophozoites on the biofilm), indicating degraded areas. Data are representative of two independent experiments, done in triplicates. At least 10 fields were assessed in each experiment. Scale bar represents 10 µm. **E** Electron microscopy images of trophozoites (in dark grey) entering and embedded within the biofilm. Lower panel: Heat map of upper panel arranged by height (red to blue) illustrating trophozoites embedded within the biofilm. Data is representative of three independent experiments, Data are representative of two independent experiments, done in triplicates. At least 10 fields were assessed in each experiment. Scale bar represents 20 µm.

**Fig. 3:**
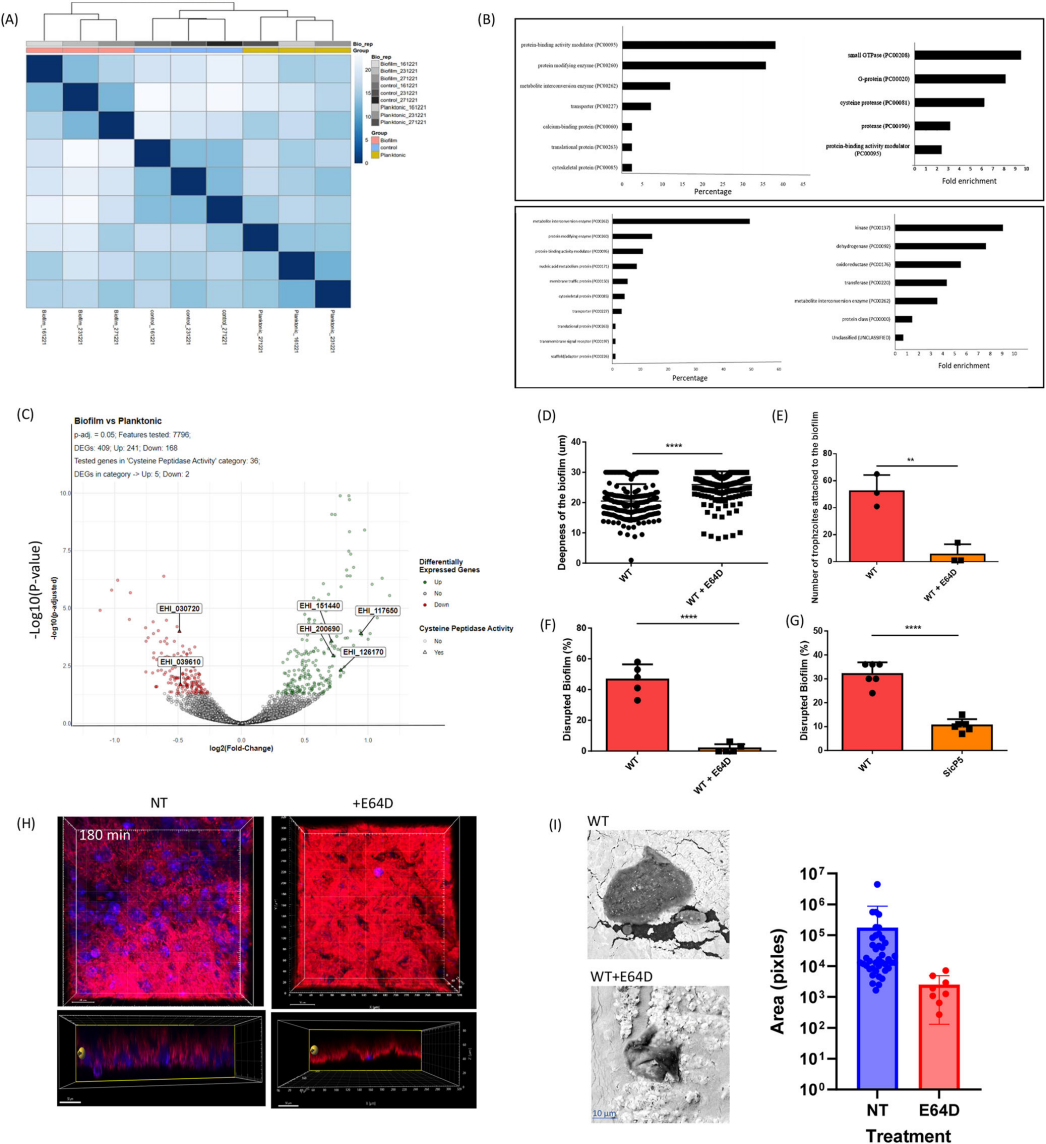
CPs promote digestive exophagy of *B. subtilis* biofilm by *E. histolytica*. **A** Heatmap illustrating the transcriptomic results, displaying the Euclidean distance between control trophozoites (control_number) and trophozoites incubated with either *B. subtilis* planktonic cells (Planktonic_number) or biofilms (Biofilm_number). Corresponds to the RNA batch that has been analyzed by transcriptomics. Darker colors indicate a stronger correlation. **B** PANTHER functional classification categories (Left panels) and statistical overrepresentation test (right panels). The Upper panels show the upregulated and the lower panels show the downregulated genes in trophozoites exposed to biofilm vs planktonic form of *B. subtilis*. **C** Volcano Plot Analysis of Differentially Expressed Genes in *E. histolytica*: Biofilm vs. Planktonic Form of *B. subtilis*, with emphasis on CPs genes based on Gene Ontology (GO) terms. Red dots represent significantly downregulated genes between the indicated treatments, while green spots represent significantly upregulated genes. Light gray dots correspond to genes that do not show a significant impact. Triangles denote genes with CPs activity according to GO and are labeled with their corresponding accession numbers. **D** Depth of trophozoites within the biofilm, whether treated with E64D or untreated, following a 3-h incubation period. Statistical analysis was performed using an unpaired T-test, and significance levels are denoted by **** indicating a *p*-value of <0.0001. The presented data represents the average results obtained from three independent biological replicates. **E** The binding capacity of viable trophozoites treated with E64D to *B. subtilis* biofilm was impaired. The percentage (%) of DAPI-stained trophozoites on the biofilm surface was assessed after incubation for 30 min at 37 °C. Trophozoites that were not treated with E64D served as the control group. T-test was performed, **p < 0.01. Data represent the average results from three biological replicates. **F** The degradation of *B. subtilis* biofilm was evaluated using control trophozoites (WT) or E64D-treated trophozoites (WT + E64D), following the methods outlined in the Materials and Methods section. Statistical analysis was performed using an unpaired T-test, and significance levels are denoted by **** indicating a *p*-value of <0.0001. The presented data represents the average results obtained from three independent biological replicates. **G** The degradation of *B. subtilis* biofilm was assessed using control trophozoites (WT) or CP5-silenced trophozoites (siCP5), following the experimental protocols outlined in the Materials and Methods section. Statistical analysis was performed using an unpaired T-test, and significance levels are denoted by **** indicating a *p*-value of <0.0001. The presented data represents the average results obtained from three independent biological replicates. **H** Confocal microscopy (at 30X magnification) was performed to examine *B. subtilis* biofilm expressing TasA-mCherry after 180 min of incubation at 37 °C with *E. histolytica* trophozoites (stained with DAPI), with and without E64D treatment. The upper panels represent the top view, while the lower panels show the side view of the biofilm. **I** Left panel shows scanning Electron Microscopy of non-treated trophozoites (NT) or E64D-treated trophozoites. Right panel shows the quantification of the area of cracked areas in the indicated backgrounds from scanning electron microscopy with ImageJ software (*n* = 5 fields). Crack size was analyzed using ImageJ software. Data are representative of two independent experiments, performed in triplicates. Less cracks were observed with an E64D treatment resulting in a smaller sample size.

To initiate the investigation into the interaction between *E. histolytica* trophozoites and *B. subtilis* biofilms, we examined the binding of trophozoites to the biofilm over time. GFP-labeled trophozoites were incubated with *B. subtilis* biofilms. By measuring the level of GFP we were able to know the quantity of trophozoites that have bound to the biofilm surface. Our observations revealed that the number of trophozoites bound to the biofilm progressively increased, eventually reaching a plateau after 30 min (Fig. 1A). To assess the degradation of *B. subtilis* biofilm by *E. histolytica* trophozoites, increasing number of trophozoites were incubated with *B. subtilis* biofilm expressing GFP for different durations. The decrease in GFP signal served as an indicator of biofilm degradation. The interaction between trophozoites and the biofilm resulted in a time and dose-dependent degradation of the biofilm. After 3 h of incubation, the biofilm exhibited a 55% degradation, and this degradation increased significantly to 79% after 6 h of incubation with 10^6^ trophozoites (Fig. 1B). Moreover, incubation with 10^5^ and 5 × 10^5^ trophozoites for 3 h resulted in a degradation of 28% and 47% of the biofilm, respectively, with the higher trophozoites concentration resulting in an increased degradation rate of 55% (Fig. 1C). Based on these findings, an incubation time of 3 h and a trophozoites concentration of 10^6^ were selected as the optimal conditions for subsequent biofilm degradation experiments. Importantly, the involvement of living trophozoites was essential for the biofilm degradation process, as paraformaldehyde-treated parasites, which are metabolically inert, did not cause any biofilm degradation (Supplementary Fig. 1). The degradation of *B. subtilis* biofilm by *E. histolytica* trophozoites was confirmed through the utilization of confocal microscopy and Scanning Electron Microscopy (SEM). Confocal microscopy revealed the formation of biofilm-cleared zones in proximity to the localizations of trophozoites, as shown in Fig. 2A. Trophozoites were observed at various levels within the biofilm, indicating their penetration and colonization throughout the biofilm structure, as depicted in Figs. 2B and 3H. Furthermore, evidence of phagocytosis was observed, with TasA-expressing cells found within the trophozoites throughout the biofilm, as illustrated in Fig. 2C. Moreover, SEM provided visual evidence of trophozoites firmly attached to the biofilm surface, as displayed in Fig. 2D, E. Notably, distinct cracks were observed beneath the trophozoites, providing compelling evidence of their active engagement in the degradation process of the biofilm. In conclusion, the combined findings from confocal microscopy and SEM confirm the degradation of *B. subtilis* biofilm by *E. histolytica* trophozoites.

### The transcriptome architecture reflects specific recognition of bacterial cells in biofilms

To investigate the impact of biofilm degradation on the parasite’s transcriptome, we utilized RNA sequencing (RNA-seq) analysis. Comparative analysis was conducted under three conditions: wild-type trophozoites (WT) as a control group, WT trophozoites incubated with planktonic *B. subtilis* (pB), and WT trophozoites incubated with *B. subtilis* biofilm (bB). In comparison to the untreated control, the presence of planktonic cells resulted in the induction of 157 transcripts and the repression of 199 transcripts (Supplementary Table 1). However, biofilms induced the expression of more transcripts (515) and repressed 543 transcripts when compared to the control group (Supplementary Table 1). This distinction in transcriptome profiles highlights significant differences between trophozoites interacting with biofilms and those interacting with planktonic cells, as illustrated in Fig. 3A and Supplementary Fig. 2. These findings suggest that trophozoites respond differently at the transcriptional level to biofilm cells compared to planktonic cells, indicating that biofilm structure may possess distinct features recognized by the parasites.

In order to evaluate the discriminatory ability of *E. histolytica* in distinguishing between *B. subtilis* biofilm cells and planktonic cells, an extensive analysis of gene expression was undertaken. Differentially regulated genes between WT+ bB (wild-type plus biofilm) and WT+ pB (wild-type plus planktonic) were categorized based on their encoded protein classes, employing PANTHER, a bioinformatics tool^30^, shown in Fig. 3B. Among the upregulated genes, the most prevalent protein classes are protein-binding activity modulators (PC00095), including Rho family GTPase (EHI_180430), protein-modifying enzymes (PC00260), represented by cysteine proteinase A-4 (EHI_050570) and metabolite interconversion enzymes (PC00262), exemplified by Lysozyme-related protein (EHI_015250). Notably, the PANTHER statistical overrepresentation test^31^ revealed a significant enrichment of genes encoding small GTPases (PC00208), like AIG1 family protein (EHI_15250), and cysteine proteases (CPs) (PC00081), such as CP (EHI_151440), among the upregulated genes in WT+ bB compared to WT+ pB. The upregulation of specific CPs expression in the parasite exposed to *B. subtilis* biofilms strongly suggests their significant role in their degradation. Conversely, the functional classification categories of the downregulated genes in WT+ bB compared to WT+ pB are displayed in Fig. 3B. The most abundant protein class among the downregulated genes is protein-modifying enzymes (PC00260), exemplified by thioredoxin reductase (EHI_155440), followed by metabolite interconversion enzymes (PC00262), including malate dehydrogenase (EHI_092450). Furthermore, the PANTHER statistical overrepresentation test indicated a significant enrichment of genes encoding kinases (PC00137), such as Pyruvate, phosphate dikinase (EHI_009530), and dehydrogenases (PC00092), exemplified by NAD(FAD)-dependent dehydrogenase (EHI_099700), among the downregulated genes in WT+ bB compared to WT+ pB. The downregulation of key redox enzyme expression in the parasite exposed to *B. subtilis* biofilms strongly suggests their role in protecting the parasite against oxidative stress. Additional comparisons, such as WT+ pB versus WT and WT+ bB versus WT, were also analyzed, and the results are presented in Supplementary Figs. 3 and 4, respectively.

### A role for CPs in the digestive exophagy and subsequent predation of *B. subtilis* biofilm cells by *E. histolytica*

CPs genes such as EHI_151440, EHI_200690, EHI_117650, and EHI_126170 were found to be upregulated in *E. histolytica* trophozoites when exposed to *B. subtilis* biofilms (Supplementary Table 1, Fig. 3C). These findings suggest the potential role of the CPs in biofilm degradation. To investigate the involvement of CPs activity in the digestion of biofilm by *E. histolytica*, trophozoites were treated with the cell-permeable CPs inhibitor, E64D (10 µM^32^), for 24 h. The incubation of trophozoites with E64D resulted in a strong inhibition of CPs activity (Supplementary Fig. 5). However, the viability of trophozoites after 24 h of treatment was only slightly affected, with approximately 65% of the trophozoites remaining viable (Supplementary Fig. 6). It is important to note that the same number of livings trophozoites were incubated with the biofilm under all conditions. The ability of E64D-treated trophozoites to attach, penetrate and degrade *B. subtilis* biofilms was severely impaired showing the important role of the CPs in biofilm degradation (Fig. 3D–F, H, I and Supplementary Fig. 7). One of the most important CPs is EhCP5 (EHI_168240), a major virulent factor that is present on the surface and secreted by the parasite^33^. EhCP5 does not show increased expression in trophozoites exposed to *B. subtilis* biofilms. However, its localization on the parasite’s surface and its secretion^34^ suggests its potential involvement in the initial phases of biofilm degradation. To test this hypothesis, we utilized RNA interference gene silencing^35^ to downregulate EhCP5 expression. EhCP5-silenced trophozoites exhibited significantly reduced CPs activity (60% less activity) and a diminished ability to degrade *B. subtilis* biofilm (70% less degradation) compared to the wild-type parasite (Fig. 3G and Supplementary Fig. 8). These findings provide strong evidence for the role of *E. histolytica* CPs, particularly EhCP5, in the degradation of *B. subtilis* biofilm.

### The interaction with *E. histolytica* alters the response of biofilm cells to antibiotics

**Fig. 4:**
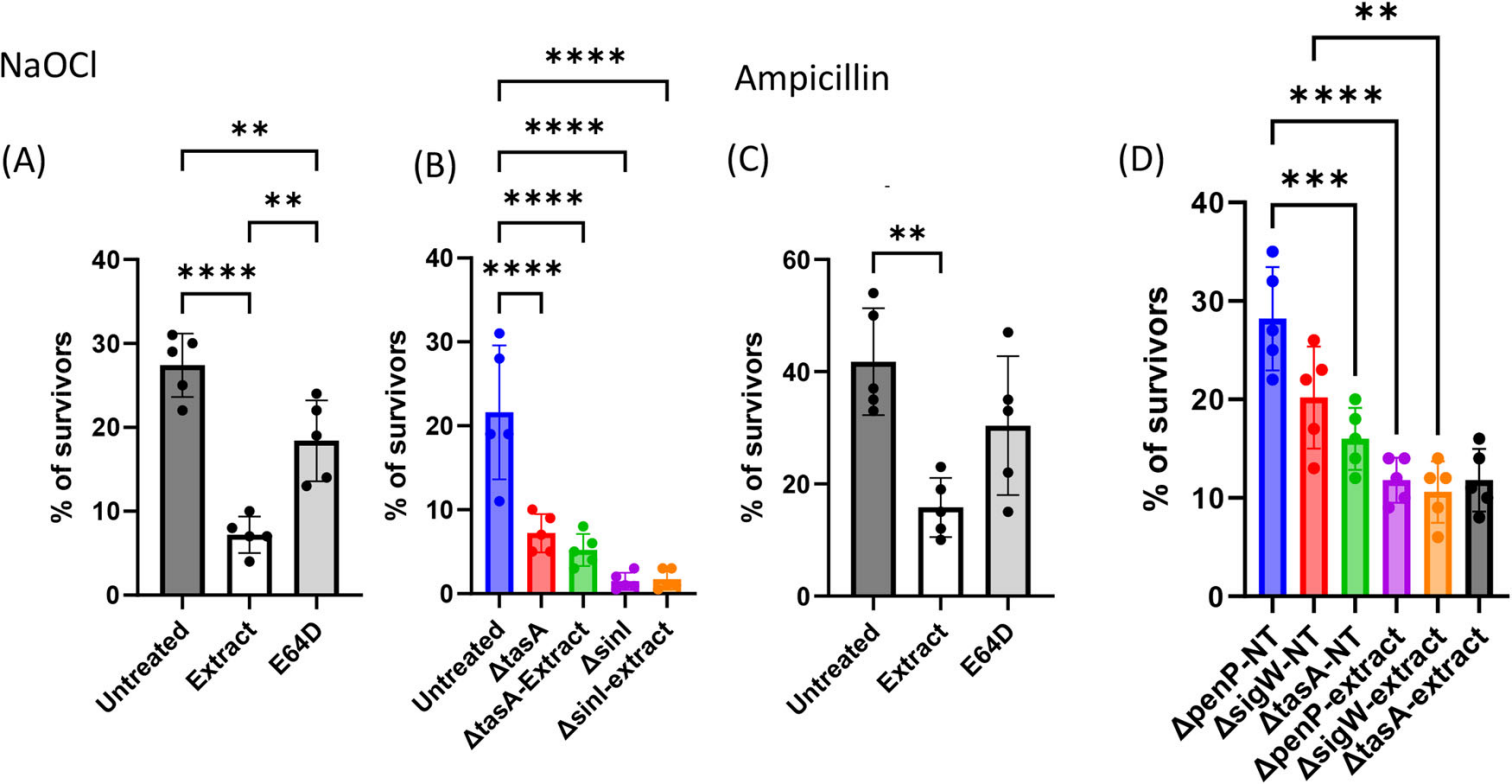
CPs from *E. histolytica* alter the antimicrobial sensitivity of *B. subtilis* biofilm cells. To determine the susceptibility to sodium hypochlorite or ampicillin within a biofilm, cell-number percentage of CFU without or with chemical stress was compared as described in materials and methods. The percentage of surviving CFU is represented by the ratio of biofilm cells treated by the sterilizing agents compared to untreated (PBS) cells or to untreated (PBS) cells for the controls. **A**, **B** CFU analysis following combinatorial treatment of sodium hypochlorite and the extract. **C**, **D** CFU analysis following combinatorial treatment of ampicillin and the extract. Graphs represent the mean ± SD from three biological repeats (*n* = 5). All Statistical analysis was performed using Brown-Forsythe and Welch’s ANOVA with Dunnett’s T3 multiple comparisons test. *p* < 0.05 was considered statistically significant.

Previous reports have indicated that the degradation of biofilms by extracellular proteases could enhance the permeability of antimicrobial agents and antibiotics within the biofilm^36^. To initiate an investigation into the effects of biofilm degradation on antibiotic resistance, trophozoites, both treated and untreated with E64D, were lysed and subsequently incubated with pre-established biofilms. The extract alone showed no toxicity to biofilm cells (Supplementary Fig. 9), and the growth of biofilm cells was not affected by dispersal agents targeting the extracellular matrix^37^. To examine the impact of biofilm dissolution on antibiotic sensitivity, the treated biofilm cells were separately exposed to two different antimicrobial agents, sodium hypochlorite (NaOCl) and the β-lactam antibiotic ampicillin, as previously conducted by our team^23,25,38^. In accordance with its matrix-targeting effect, treatment of pre-established biofilms with extracts from trophozoites noticeably amplified the response of biofilm cells to NaOCl (Fig. 4A). Furthermore, the partial reversal of this synergistic effect was observed upon inhibition of CPs activity in the trophozoite extracts (Fig. 4A).

The sensitivity of the *tasA* mutant biofilm to NaOCl was assessed both with and without treatment with amoeba extract. No discernible difference in the mutant biofilm’s response to NaOCl exposure was observed across the various treatments (Fig. 4B). This finding underscores the significance of TasA in promoting antibacterial resistance to NaOCl. Deletion of the master regulator SinI, which represses both exopolysaccharide and TasA formation^39^, resulted in slightly heightened sensitivity to NaOCl compared to the TasA mutant. This suggests that the presence of residual exopolysaccharides in the TasA mutant offers some degree of resistance to biofilm cells. No additional effect was observed when the mutants were combined with the extract, in contrast to the single untreated mutant (Fig. 4B). Hence, the presence of the extracellular matrix is essential for the extract to influence the sensitivity of biofilm cells to sodium hypochlorite.

Furthermore, *E. histolytica*’s CPs are responsible for a significant increase in the sensitivity of biofilm cells to high doses of ampicillin, reaching 80% of the minimum biofilm inhibitory concentration (MBIC) (Bucher and Kolodkin-Gal, unpublished results) (Fig. 4C). In contrast, the *tasA* mutant biofilm demonstrated an increased sensitivity to ampicillin. This sensitivity did not decrease when exposed to the extract, highlighting the importance of the extracellular matrix for antibiotic resistance (Fig. 4D). In *B. subtilis*, resistance to ampicillin is conferred through the activation of the general cell wall stress response, regulated by the alternative extracytoplasmic sigma factor SigW^40,41^. Consistently, the *sigW* mutant displayed heightened sensitivity to ampicillin, yet it exhibited a significant response to the extract (Fig. 4D). In addition, ampicillin can be degraded by the β-lactamase PenP, which is expressed from a SigW-dependent promoter and during biofilm formation^42^. Similarly, a *penP* mutant, akin to the *sigW* mutant, exhibited increased sensitivity to ampicillin but responded significantly to the extract. These findings indicate that incubation with lysate from WT amoeba effectively restored sensitivity to ampicillin by targeting the extracellular matrix.

### The role of the bacterial matrix proteins in prey-predator interactions with *E. histolytica* trophozoites

**Fig. 5:**
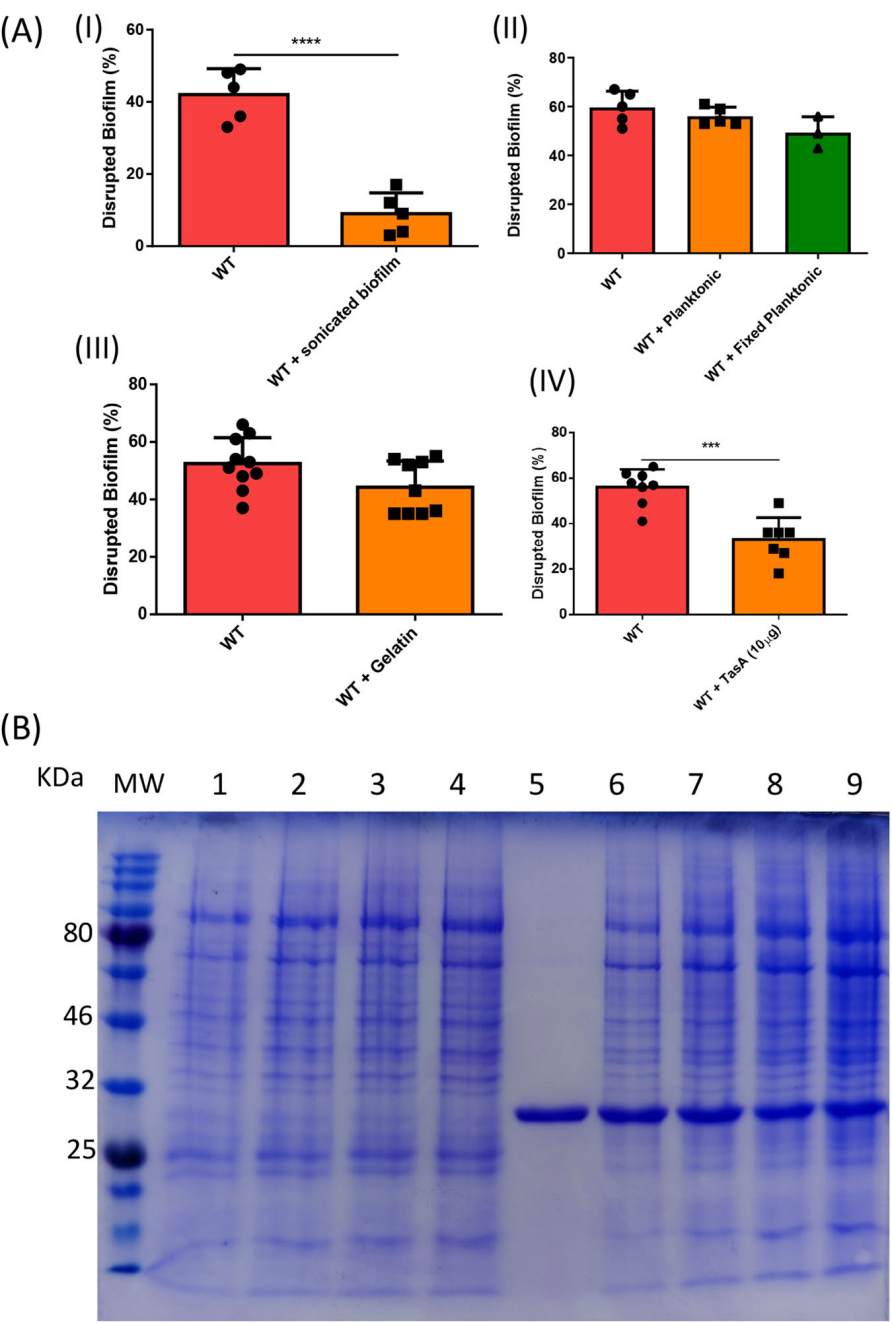
The role of extracellular matrix protein TasA in the predation of biofilm cells. **A** Biofilm degradation assay with different competition. Unpaired T-test was used for all experiments, ***p < 0.001 and ****p *<* 0.0001). Data represent averages of results from three biological replicates. I: Degradation of *B. subtilis* by *E. histolytica* trophozoites (WT) and trophozoites incubated with sonicated *B. subtilis* biofilm (WT+sonicated biofilm). II: Degradation of *B. subtilis* by *E. histolytica* trophozoites (WT) and trophozoites incubated with *B. subtilis* planktonic form (WT+Planktonic) and fixed *B. subtilis* planktonic form (WT+Planktonic fixed). III: Degradation of *B. subtilis* by *E. histolytica* trophozoites (WT) and trophozoites incubated with gelatin (1%). IV: Degradation of *B. subtilis* by *E. histolytica* trophozoites (WT) and trophozoites incubated with TasA (0.01%). Data represent averages of results from three biological replicates. **B** TasA degradation by *E. histolytica* total lysate. (1) Lysate of *E.histolytica* wild-type trophozoites (lysate WT), (2) TasA (2 μg) + 10 μg lysate WT, (3) TasA (2 μg)+ 15 μg lysate WT, (4) TasA (2 μg) + 20 μg lysate WT, (5) TasA (2 μg), (6) TasA (2 μg) + 5 μg of lysate from E64D (10 μM) treated trophozoites (lysate E64D), (7) TasA (2 μg)+ 10 μg of lysate E64D, (8) TasA (2 μg)+ 15 μg of lysate E64D, (9) TasA (2 μg)+ 20 μg of lysate E64D. TasA was incubated for 3 h at 37 °C. The degradation of TasA was analyzed by SDS-PAGE following Coomassie staining.

The molecular mechanisms involved in the binding of *E. histolytica* to mammalian cells and planktonic bacteria have been extensively investigated through competition experiments using low-molecular-weight carbohydrates^43,44^. Building upon this established methodology, we employed the same experimental approach to explore the binding mechanism of *E. histolytica* trophozoites to *B. subtilis* biofilm. The involvement of the Gal/GalNac receptor on the surface of *E.histolytica*^45^ in the degradation of *B. subtilis* biofilm was investigated by comparing the biofilm degradation between trophozoites incubated with and without galactose (2%) (Supplementary Fig. 10A). Surprisingly, no significant difference in biofilm degradation was observed when the biofilm was incubated with or without galactose, suggesting that the Gal/GalNac receptor is not involved in this process. Similarly, the addition of mannose (1%) or asialofetuin (0.05%) (a glycoprotein that is found in the blood serum of various animal species and commonly used to study specific interactions between glycoproteins and cell surface receptors^46^) did not show a significant difference in the degradation of *B. subtilis* biofilm by trophozoites (Supplementary Figs. 10B, C). Furthermore, the planktonic form of *B*. subtilis did not impair the degradation of *B. subtilis* biofilms by *E. histolytica* (Fig. 5AII), while sonicated biofilms acted as potent inhibitors of parasite binding (Fig. 5AI). These findings suggest that the parasite is binding to a specific component within the biofilm. To determine if TasA is directly involved in the binding of *E. histolytica* to the biofilm, pure TasA (0.01%) was used as a competitor, while gelatin (1%) served as a protein control for TasA. Gelatin did not impair biofilm degradation by *E. histolytica*, indicating it does not compete with the parasite or contribute to the binding process (Fig. 5A III). However, purified TasA significantly reduced the ability of the parasite to attach to the biofilm (Fig. 5 AIV), strongly suggesting that TasA is involved in the binding between *E. histolytica* and the biofilm. Although CP-dependent TasA degradation was observed, purified TasA did not affect the secretion of CPs by the parasites (Fig. 5B and Supplementary Fig. 11). The degradation of TasA is also consistent with the reduced response of *tasA* mutant to the combinatory effect of parasite CPs and antimicrobials (Fig. 4B and D). Collectively, these findings suggest that TasA plays a dual role in the predator-prey interaction with *E. histolytica*, serving as both the ligand for parasite binding and a target for CPs-mediated degradation.

### The interaction with *B. subtilis* biofilms protects *E. histolytica* against oxidative stress and regulates its cytopathic activity

**Fig. 6:**
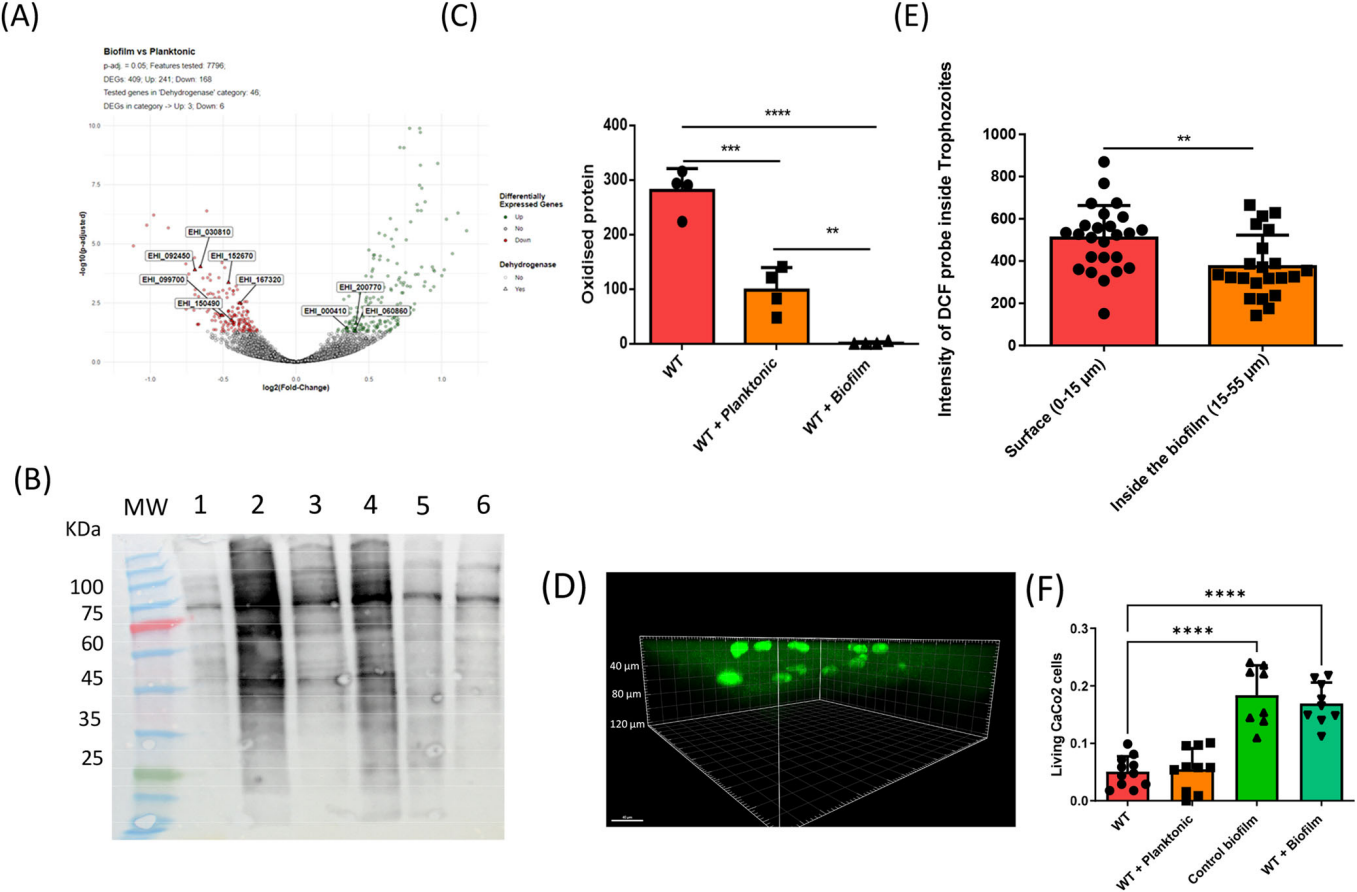
The biofilm provides protection to *E. histolytica* against OS and prevents the degradation of CaCo2 cells by the parasite. **A** Volcano Plot Analysis of Differentially Expressed Genes in *E. histolytica*: Biofilm vs. Planktonic Form of *B. subtilis*, with Emphasis on Dehydrogenase-Related Genes Based on Gene Ontology (GO) Term. In the visualization, the red dots indicate genes that are significantly downregulated under the indicated treatments, while the green spots represent genes that are significantly upregulated. Genes that do not show a significant impact are depicted as light gray dots. Notably, the genes represented with triangles denote those with dehydrogenase activity based on Gene Ontology (GO) annotations, and they are labeled with their corresponding accession numbers. **B** Trophozoites were incubated with either the planktonic or biofilm form of *B. subtilis* and subsequently exposed to H_2_O_2_ (2.5 mM, 30 min). The quantification of oxidized proteins in the trophozoites was performed using the OxyBlot method. (1) WT, (2) WT + H_2_O_2_, (3) WT + Planktonic, (4) WT + Planktonic + H_2_O_2_, (5) WT + Biofilm and (6) WT + Biofilm + H_2_O_2_. An uncropped gel is provided in Supplementary Fig. 14. **C** Graphic representation of the data from the Oxyblot analysis. The data have been normalized using the total protein normalization method. An unpaired T-test was used, **p < 0.01, ***p < 0.001, ****p < 0.0001. Data represent averages of results from four biological replicates. **D** Confocal microscopy (X30) of *B. subtilis* NCIB3610 biofilm cells carrying TasA-mCherry after 180 min incubation with trophozoites (stained with DAPI) at 37 °C and exposed to H_2_O_2_ (0.5 mM, 10 min). The trophozoites inside the biofilm were stained with the probe H2DCFDA, resulting in a green fluorescence that indicates the presence and levels of reactive oxygen species (ROS) inside each trophozoite. The depth of each trophozoite within the biofilm was also recorded. **E** The fluorescence intensity level of H2DCFDA was compared between trophozoites according to their depth in the biofilm. Trophozoites on the surface (above 15 µm) and trophozoites located deeper within the biofilm (below 15 µm). Statistical analysis was performed using an unpaired T-test, with significance denoted by ** indicating a *p*-value of <0.01. The presented data represents the average results obtained from two biological replicates. **F** The cytopathic activity of *E. histolytica* trophozoites on Caco-2 cells was evaluated after incubation with either planktonic *B. subtilis* cells or *B. subtilis* biofilm. Statistical analysis was performed using a one-way ANOVA test, with significance levels denoted as **** for a *p*-value <0.0001. The data presented represents the average results obtained from three independent biological replicates.

Our previous work supports the role of bacterial metabolites in protecting *E. histolytica* against reactive oxygen species (ROS) (for a recent review see ref. ^5^). We observed that *E. histolytica* trophozoites penetrate biofilms and become embedded within them (Figs. 2B, 3D, H). These findings raise a hypothesis that the parasites use the biofilm as a protective layer, potentially against ROS. Several genes involved in *E. histolytica* response to oxidative stress (OS) like thioredoxin reductase (EHI_155440)^47^, EhNO1 (EHI_110520) (EhNO1 is mainly involved in ferric reduction)^48^, the DNA damage recognition like the excision repair protein RAD23 (EHI_001400)^49^ and the levels of many dehydrogenases (Supplementary Tables 1 and 2, Figs. 3B and 6A) were downregulated in trophozoites exposed to biofilms. To test the hypothesis that biofilms serve as a protective layer versys ROS, two methods were employed. The first method involved quantifying the levels of oxidized trophozoite proteins. Trophozoites were subjected to three conditions: incubation alone as a control, incubation with the planktonic form of *B. subtilis*, and incubation with the biofilm form of *B. subtilis*. Subsequently, all trophozoites were exposed to 2.5 mM hydrogen peroxide (H_2_O_2_) for 30 min. The total amount of oxidized proteins (OXs) in the parasite was determined by immunoblot detection of carbonyl groups introduced into proteins by oxidative stress (OS) (Oxyblot). Notably, in the presence of H_2_O_2_ (2.5 mM), the control trophozoites exhibited significantly higher levels of OXs compared to both the trophozoites incubated with the planktonic form and those incubated with the biofilm form of *B. subtilis* prior to exposure to H_2_O_2_ (Fig. 6B, C). Furthermore, trophozoites incubated with the biofilm form of *B. subtilis* demonstrated a significant decrease in OXs compared to both the control trophozoites and those incubated with the planktonic form, thus indicating the protective role of biofilm against H_2_O_2_-induced oxidative stress (Fig. 6B, C). The second method utilized a confocal microscope to observe the localization of trophozoites containing ROS within different layers of the *B. subtilis* biofilm. These trophozoites were incubated with H_2_O_2_ (0.5 mM, 10 min), and the presence of ROS inside them was detected using H2DCFDA, a cell-permeant fluorescein-based indicator commonly used for ROS detection in cells^50^. To ensure optimal detection of ROS using the H2DCFDA reagent in cell imaging, we employed a lower concentration of H_2_O_2_ and reduced incubation time for the confocal microscopy compared to the Oxyblot assay. This adjustment was made considering the sensitivity of the H2DCFDA reagent in detecting ROS within the cells. Our findings revealed that trophozoites of *E. histolytica* located at the top of the biofilm exhibited higher levels of ROS compared to those that had penetrated the lower layers (Fig. 6D, E). Based on the observation that, at the same level within the biofilm, some parasites exhibit a strong ROS signal, while others do not, we can conclude that the H2DCFDA reagent penetrates deeply into the lower layers of the biofilm (Fig. 6D). These results suggest that the parasite’s ability to penetrate the biofilm serves as a strategy to evade ROS, which is consistent with the existence of oxygen gradients within microbial biofilms^40,41^.

*E. histolytica* is known for its ability to damage the mucus layer of the large intestine, a critical step in its pathogenicity^51^. The presence of bacterial biofilms within this mucus layer^52^ raises intriguing questions about whether these biofilms contribute to the parasite’s capacity to reach and destroy intestinal epithelial cells. To investigate this hypothesis, we performed a cytopathic assay to assess the ability of *E. histolytica* to destroy a monolayer of CaCo2 cells covered with a biofilm of *B. subtilis* (Fig. 6F). The results revealed that the *B. subtilis* biofilm effectively prevented *E. histolytica* trophozoites from damaging the CaCo2 cells, whereas planktonic *B. subtilis* did not exhibit this protective effect. These findings strongly suggest that the *B. subtilis* biofilm functions as a shielding barrier for mammalian cells, potentially impeding the parasite’s progression toward them (Fig. 6F).

### Digestive exophagy by CPs is also observed during *E. coli–E. histolytica* interactions

**Fig. 7:**
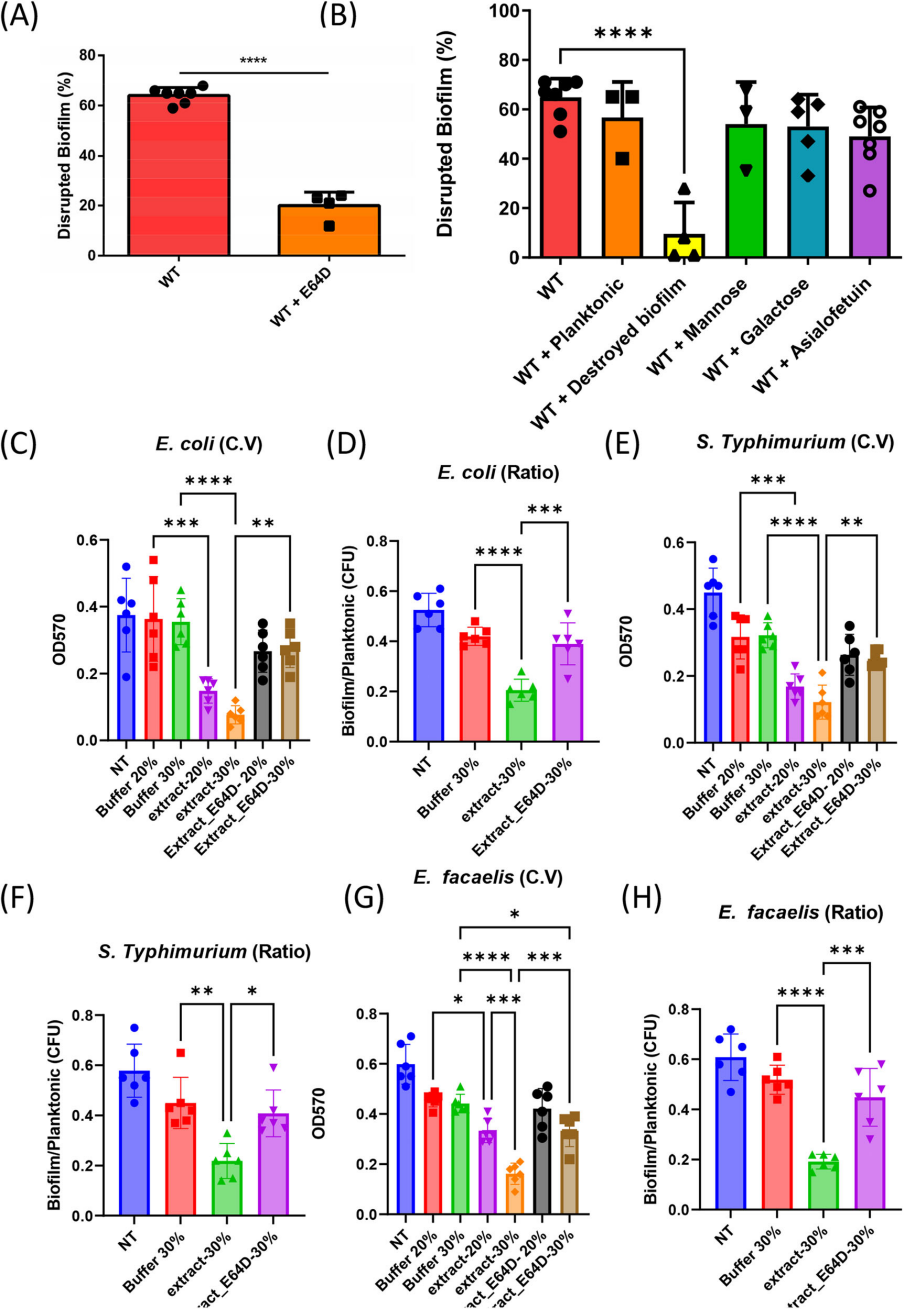
*E. histolytica* trophozoites actively degrade biofilms formed by enteric bacteria. **A** The degradation of *E. coli* biofilm was assessed after 1 h of incubation with control trophozoites (WT) or E64D-treated trophozoites (WT + E64D). Statistical analysis using an unpaired T-test revealed a significant difference, with **** indicating a *p*-value of <0.0001. The presented data represents the average results obtained from three biological replicates. **B** The degradation of *E. coli* biofilm was evaluated using *E. histolytica* trophozoites (WT) and trophozoites incubated with different conditions: *E. coli* planktonic form (WT+planktonic), disrupted *E. coli* biofilm (WT + disrupted biofilm), mannose (1%) (WT + Mannose), galactose (2%) (WT + Galactose), and asialofetuin (0.05%) (WT + Asialofetuin). Statistical analysis using an unpaired t-test revealed significance levels denoted as * for a *p*-value <0.05 and **** for a *p*-value <0.0001. The presented data represents the average results obtained from three biological replicates. **C** Crystal violet assay was performed on *E. coli* MG1655 cells were diluted 1:100 into a fresh TSB. 100 μl of cultures were split into a 96-well polystyrene plate and further incubated at 37 °C. Following overnight growth biofilms were treated as described in materials and methods, and crystal violet assay assessed the biofilm formation. Graph represents the mean ± SD from two biological repeats (*n* = 6). All Statistical analysis was performed using Brown-Forsythe and Welch’s ANOVA with Dunnett’s T3 multiple comparisons test. *p* < 0.05 was considered statistically significant. **D** *E. coli* MG1655 cells were diluted 1:100 into a fresh TSB. 100 μl of cultures were split into a 96-well polystyrene plate and further incubated at 37 °C. Following overnight growth biofilms were treated as described in materials and methods, and CFU assay assessed the biofilm biomass and planktonic biomass. Graph represents the mean ± SD from two biological repeats (*n* = 6). All Statistical analysis was performed using Brown-Forsythe and Welch’s ANOVA with Dunnett’s T3 multiple comparisons test. *p* < 0.05 was considered statistically significant. **E** Crystal violet assay was performed on *S. typhimurium* cells as in (**D**). **F** Biofilm/planktonic ratio assay was performed on *S. typhimurium* cells as in (**E**). **G** Crystal violet assay was performed on *E. faecalis* cells as in (**D**). **H** Biofilm/planktonic ratio assay was performed on *E. faecalis* cells as in (**E**).

**Fig. 8:**
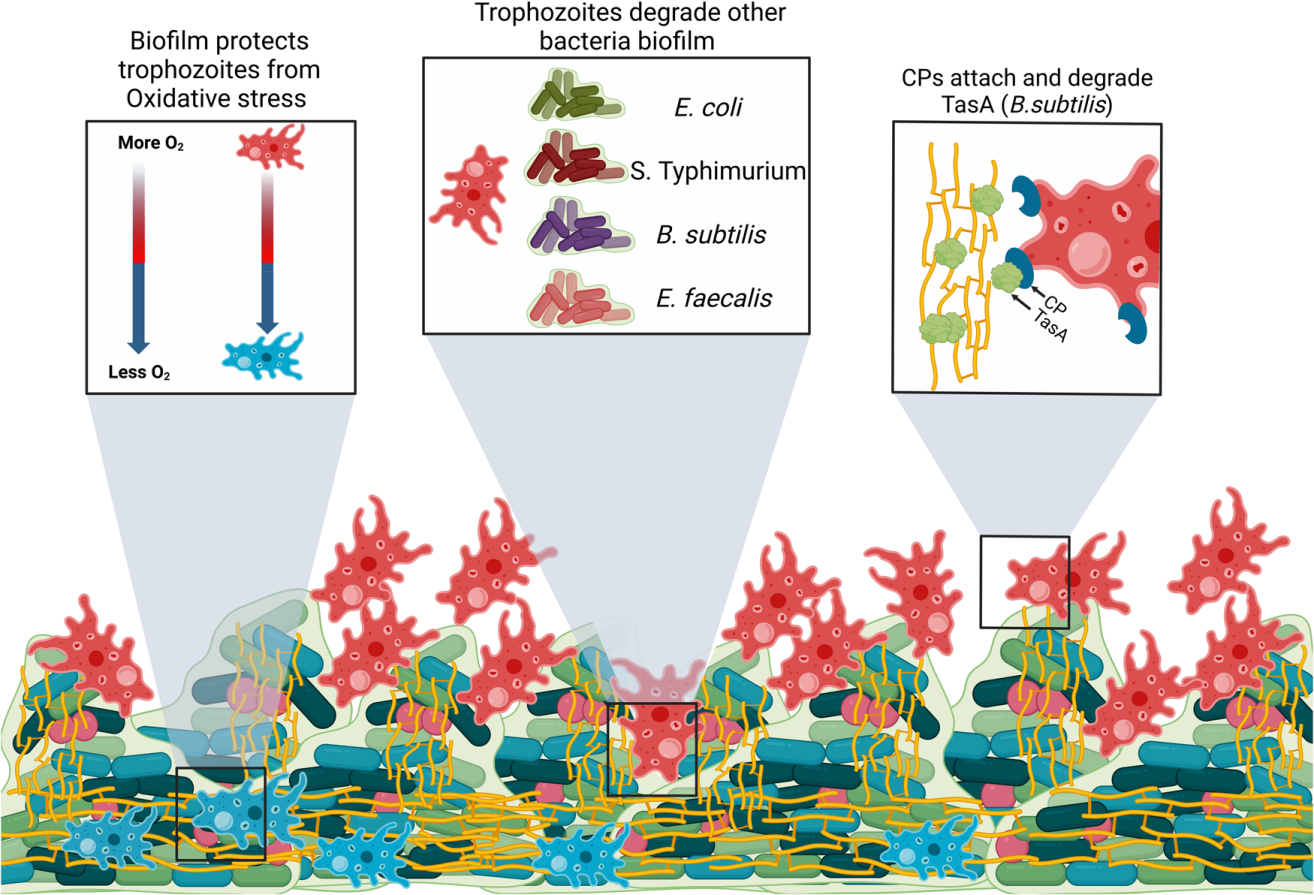
A model summarizing biofilm-parasite interaction. The Figure was created with BioRender.com (agreement number AP25K9KR4A*)*.

*E. histolytica* is exposed to a complex bacterial flora in the gut, including biofilm-forming bacteria like *E. coli*^53^. Our findings that EhCPs can degrade *B. subtilis* biofilm, and the proteinous nature of both *E. coli* and *B. subtilis* biofilms, led us to test whether EhCPs can degrade *E. coli* biofilm. Our data indicate that *E. histolytica* trophozoites are able to degrade *E. coli* biofilm (Fig. 7A). In contrast, E64D-treated-trophozoites were unable to degrade *E. coli* biofilm (Fig. 7A). The predation of *E. coli* biofilms by the parasite was inhibited in the presence of biofilm cells and not their planktonic counterparts (Fig. 7B), indicating a specific recognition of the biofilms by the parasite. Unlike *B. subtilis*, adhesion involved a carbohydrate bacterial component as it was inhibited by the glycoprotein asialofetuin (Fig. 7B) and the matrix amyloid-forming protein CsgA was resistant to CPs (Fig. S12). To confirm that the effect of biofilm degradation was specific, trophozoites, both treated and untreated with E64D, were lysed and subsequently incubated with pre-established biofilms. We compared the biofilm degradation as judged by crystal violet^54^ of the remaining attached biomass following treatment (Fig. 7C), with the biofilm/planktonic cell counts (Fig. 7D) as done by us and others previously^55^. These results clearly indicated that the CPs dependent reduction in the biofilm biomass of *E. coli* was not due to an overall reduction of growth but rather to an alteration of the ratio between the detached (free-living) and attached cells (biofilm). To expand our investigation, we also explored the capability of *E. histolytica* to disrupt biofilms formed by other enteric bacteria. Remarkably, the trophozoite lysate exhibited the ability to contribute significantly to the degradation of *S. Typhimurium* (Fig. 7E, F) and *E. faecalis* (Fig. 7G, H) biofilms. These results indicate that CPs mediate biofilm dissolution on a broader repertoire of enteric pathogens. Since the observed effect was specifically targeted at the biofilm biomass (Fig. 7D, F, H), it is intriguing to speculate that this could be attributed to a broad-spectrum impact on the various adhesins that make up the microbial matrix (Fig. 8).

## Discussion

In the GI, bacteria reside as biofilm or structured aggregates. Therefore, it is expected that the binding of *E. histolytica* trophozoites to the biofilm represent the first step in the biofilm degradation process followed by biofilm degradation. These interactions may affect probiotic biofilms, as well as biofilms formed by pathogenic bacteria during co-infection. Our data suggests that CPs may play a role in binding. One potential candidate for the binding of trophozoites on biofilm is a 112 kDa adhesin consisting of two polypeptides, one weighing 49 kDa and the other weighing 75 kDa. The former exhibits CP activity, while the latter demonstrates adhesin activity (for a review see ref. ^56^). Furthermore, our findings indicate that TasA has the ability to inhibit the binding of the parasite to *B. subtilis* biofilm. This observation suggests the presence of a TasA-binding protein on the surface of *E. histolytica*. TasA serves both as a substrate for trophozoite binding and a target of CPs, offering a significant role for matrix proteins in parasite-biofilm interactions. CPs are essential virulence factors of *E. histolytica* responsible for the degradation of mucus and extracellular matrix components such as collagen, fibronectin, and immunoglobulins^57^. Our data strongly support the crucial involvement of parasite CPs in the degradation of biofilms, facilitating the interaction between parasites and biofilm cells through the process of digestive exophagy^58^. Our work also demonstrates that EhCP5 is directly involved in the degradation of *B. subtilis* biofilms and is potentially supported by EhCP4 and EhCP6 as their expression is upregulated following exposure to biofilms. The significant role of EhCP5 in biofilm degradation, even in the absence of a concurrent increase in its mRNA expression, implies that a similar scenario might apply to other EhCPs within the extensive repertoire of 35 EhCPs identified in the parasite’s genome^59^. This underscores the importance of adopting complementary proteomic approaches to investigate the involvement of EhCPs in biofilm degradation. The specific response of the amoeba transcriptome to biofilms and the subsequent activation of digestive enzymes suggests that these interactions frequently occur in nature and contributed sufficiently to the fitness of the parasite during its evolution to establish conditional response. One methodological limitation of our experimental approach lies in its focus on mono-species biofilms, which was chosen for ease of analysis. However, it is crucial to recognize that biofilms found in natural environments comprise diverse multi-species organisms^60^. Therefore, future investigations should aim to elucidate the response of the parasite towards multi-species biofilms, as this will provide a more comprehensive understanding of its interactions within complex ecological systems.

Upon penetrating the biofilm, the parasite is protected from ROS, which promotes its survival. It is possible that *B. subtilis* biofilms provide to the parasite antioxidant compounds that are not produced by the planktonic form. Activity of tricarboxylic acid (TCA) cycle during early biofilm growth has been reported leading to the accumulation of TCA cycle intermediate including citrate, malate and oxaloacetate^61^ that are known antioxidants^62^. One of these compounds, oxaloacetate, protects the parasite against OS^63^. Another antioxidant compounds specifically produced by *B.subtilis* biofilms is the red-colored pigment pulcherrimin^64^. Future investigations should aim to elucidate the roles of oxaloacetate and pulcherrimin in protecting parasides embedded within *B.subtilis* biofilms against OS. It is also possible that the safeguarding effect against OS may be attributed to the establishment of an oxygen gradient within the biofilm, where reduced oxygen levels are observed towards its lower region^65,66^ thereby harboring parasites that exhibit lower levels of OS. The colonization of the lower part of the biofilm by *E. histolytica* trophozoites represents a novel adaptive strategy, among various others^67^, employed by the parasite to effectively mitigate OS within the GI microenvironment.

Interestingly, *B. subtilis* cells seem to also respond to the presence of the parasites. The extract application, and subsequent degradation of the matrix by the parasite significantly induced the cell wall stress response^42^, potentially due to the specific role of TasA as cell-cell adhesion^68^. CPs from the amoeba were clearly synergistic with both NaOCl and clinically relevant concentrations of ampicillin, and these results can be attributed to matrix degradation. Furthermore, our results also support a role for CPs in the degradation of biofilms formed by *E. coli*, as well as additional enteric bacteria. This suggests that the CP-mediated biofilm degradation mechanism employed by *E. histolytica* is not limited to specific bacterial species, but rather has broad implications across different pathogenic bacteria. It’s worth noting that the impact of bacterial biofilms on intestinal epithelial cell health can vary, depending on the specific types of bacteria present and the context in which the biofilm is formed^69^. In some cases, biofilms can provide a protective layer for pathogenic bacteria to evade host defense^70^. For example, an increased number of *E. coli* biofilms adhering to the intestinal lining has been linked to the development of ulcerative colitis^10^. On the other hand, biofilms in the healthy gut may have beneficial effects for the host by boosting the functions of the microbiota, such as enhancing host defense^71^ or promoting the colonization and longer persistence of beneficial bacteria in the gut mucosa, which can prevent the colonization of pathogens^72^. Our findings highlight the profound impact of parasite interactions on the fitness of microbial cells, exerting both beneficial and detrimental effects on biofilm microbiome communities^71^. These interactions can either facilitate the colonization and prolonged persistence of beneficial bacteria in the gut mucosa, thereby hindering the colonization of pathogens^72^.

The capacity of protists to degrade biofilms bears critical importance on human health^73^. The U.S. Centers for Disease Control and Prevention (CDC) has estimated that bacterial biofilms are responsible for 60% of chronic infections, including burn wounds, chronic ulcers of limbs associated with diabetes, periodontitis, osteomyelitis, chronic wounds, and cystic fibrosis lungs^74,75^. Biofilm cells are inherently more resistant to the host immune system and to antibiotics^76^. One unexplored resource for uncovering novel anti-biofilm agents with a broad spectrum are protozoan parasites^74,75^. This neglect is not trivial as single-cells eukaryotes and bacteria exert well-established predator-prey interactions^77^, which should be extendable to bacterial biofilms in various ecological niches, most notably, zooplankton grazers-phytoplankton mats interactions^78^. Our finding of specific activation of matrix degrading CPs in response to the microbial biofilm indicates that amoeba are adapted to biofilm preys, and may serve as a new unexplored reservoir of novel therapeutic approaches to treat biofilms. Furthermore, our findings here that *E. histolytica* trophozoites can use the biofilm as a shield reducing the OS of the parasite, while fundamentally altering stress/antibiotic tolerance in the remaining biofilm cells highlight biofilm-amoeba interactions as one unexpected significant regulator of stress tolerance and pathogenicity across the microbial kingdom.

## Methods

### *E. histolytica* culture

*E. histolytica* trophozoites of strain HM-1:IMSS (from Prof. Samudrala Gourinath, Jawaharlal Nehru University, New Delhi, India), were grown at 37 °C in 13 × 100 mm in screw-capped Pyrex glass tubes in serum-free Diamond’s TYI S-33 medium (Johnson and Johnson, Hyclone, USA) to the exponential phase. Trophozoites were harvested from their growth support by incubating the tubes by tapping the glass tubes followed by centrifugation (Eppendorf centrifuge 5810R, rotor A-4-62) according to a previously reported protocol^79^. *E. histolytica* trophozoites of strain HM-1:IMSS (from Prof. Samudrala Gourinath, Jawaharlal Nehru University, New Delhi, India), were grown at 37 °C in 13 × 100 mm in screw-capped Pyrex glass tubes in serum-free Diamond’s TYI S-33 medium (Johnson and Johnson, Hyclone, USA) to the exponential phase. Trophozoites were harvested from their growth support by incubating the tubes by tapping the glass tubes followed by centrifugation (Eppendorf centrifuge 5810R, rotor A-4-62) according to a previously reported protocol^79^.

### Cell cultures

Caco-2 cells were obtained from the American Type Culture Collection (HTB-37) (a gift from Dr. Shlomi, Faculty of Biology, Technion, Israel) and cultured in Dulbecco’s modified Eagle medium (DMEM) supplemented with 10% dialyzed fetal bovine serum (BioWest, S00GG), 1% penicillin-streptomycin (BioWest, MS01L9), and 4 mM glutamine (BioWest, MS01PT). The cultures were grown in 15 by 10 cm plastic tissue culture flasks and maintained in a humidified atmosphere of 5% CO_2_ at 37 °C the media was changed every 2 days^80^. The cultures were grown in 15 by 10 cm plastic tissue culture flasks and maintained in a humidified atmosphere of 5% CO_2_ at 37 °C the media was changed every 2 days^80^.

### Bacterial strains

All *B. subtilis* strains utilized in this study were derived from the proficient biofilm-forming strain NCIB3610^81^. The cell wall mutants (∆*penP*:kan, ∆*sigW*::tet) and mutants involved in the formation of the extracellular matrix (∆*tasA*::kan, ∆*sinI*::spec) were previously described^42,68^.

The *E. coli* strain used is MG1655 K12.

When indicated, *S. enterica* reservoir Typhimurium, kindly provided by Prof. Ilan Rosenshine), and *Enterococcus faecalis* 29212^82^ were used.

### Biofilm formation

#### B. subtilis

The biofilm formation were conducted using *B. subtilis* GFP-expressing strain NCIB3610, specifically the *amyE::P*_*hyperspank*_*-gfp* variant^83^. A single colony was isolated from lysogeny broth (LB) plates and grown to mid-logarithmic phase in a 3-ml LB culture, with shaking at 37 °C for 4 h at 200 rpm using a New Brunswick scientific, Innova 4300 shaker. For biofilm preparation, we followed a procedure adapted from Xiaoling Wang et al.^84^. Cells from the mid-logarithmic phase were diluted 1:10 into a serum-free Diamond’s TYI S-33 medium and grown overnight at 30 °C without shaking. The growth was carried out in 24-well plates, with each well covered by 1 ml of serum-free Diamond’s TYI S-33 agar.

In experiments involving disrupted *B. subtilis* biofilms, the biofilm formed overnight was subjected to sonication using a bioruptor UCD 200 (Diagenode). The sonication process involved three cycles of 15 s on/off at medium intensity to disrupt the biofilm.

#### E. coli

The biofilm formation was conducted using *E. coli* GFP-expressing strain k12 MG1655, generously provided by Dr. Ido Bachelet from Bar-Ilan University, Israel. A single colony was isolated from LB plates and grown to mid-logarithmic phase in a 3-ml LB broth culture. The culture was incubated for 4 h at 37 °C with shaking at 200 rpm.

To prepare the biofilms, cells from the mid-logarithmic phase were diluted 1:10 in serum-free Diamond’s TYI S-33 medium. Subsequently, the diluted cells were cultivated overnight at 37 °C in 24-well plates without shaking, allowing the biofilms to form.

In experiments where biofilm disruption was required, the biofilm was disrupted through a pipetting process that entailed gently lifting and lowering it multiple times. Given the relative weakness of *E. coli* biofilm compared to *B. subtilis*, pipetting alone proved adequate to disturb its structure.

### Biofilm degradation assay

Trophozoites (1 × 10^6^) were incubated on *B. subtilis* biofilm at 37 °C for 3 h for *B. subtilis* and 1 h for *E. coli*, without shaking. To confirm that the degradation of the biofilm was attributed to the activity of living amoebae, trophozoites were fixed with 4% paraformaldehyde for 30 min at room temperature. Subsequently, the fixed trophozoites were incubated for 3 h with *B. subtilis* biofilm and 1 h with *E. coli* biofilm. To quantify the extent of biofilm degradation, the GFP signal intensity of each well was compared to the control (biofilm incubated without trophozoites) using ImageJ software. The original pictures, containing the GFP signal, were converted into black and white image, where white represents a stronger GFP signal and black indicates a weaker signal. Therefore, a darker image indicates a higher level of biofilm destruction and a weaker biofilm. The level of black pixels in the image, which reflects the degree of biofilm degradation, was quantified and normalized based on the control sample.

### *E. histolytica* trophozoites binding to *B. subtilis*

The trophozoites used for this experiment transfected with pEhExGFP and express the protein GFP (a kind gift from Dr. Tomoyoshi Nozaki)^85^. Trophozoites (1 × 10^6^) were incubated on *B. subtilis* or *E. coli* biofilm at 37 °C for 10 to 40 min without shaking. Following the incubation period, the trophozoites were carefully removed, and the biofilm was washed once with serum-free Diamond’s TYI S-33 medium. Photographs of each biofilm were captured using the Olympus MVX10 microscope equipped with the Olympus DP73 camera, GFP laser, and 2.5x zoom. The software used for image acquisition and analysis was cellsens dimension. The quantification of GFP-labeled trophozoites on the biofilm surface was performed using automated cell counting in ImageJ. Each image was converted into a black and white format to enhance trophozoite visibility, with the trophozoites appearing as white. The “Analyze Particles” function in ImageJ was utilized, applying a size range of 100 to 3000 pixels^2^, to detect and count the trophozoites.

### Immunofluorescence inverted confocal microscopy

Trophozoites (1 × 10^6^) were incubated with *B. subtilis* biofilms expressing the matrix protein TasA fused to mCherry, which allowed us to label both the biofilm cells and their assembled matrix^68^, for either 30 min or 180 min at 37 °C. Trophozoites that did not attach were washed away using serum-free Diamond’s TYI S-33 medium. The biofilm was then fixed with paraformaldehyde (4%) for 30 min at room temperature. Subsequently, to enhance the detection and visualization of *E. histolytica* trophozoites during the imaging process, the biofilm was stained with DAPI (20 µg/ml) for 1 h at 4 °C in the dark. At the applied concentration of DAPI, the trophozoites were predominantly stained, while the bacteria within the biofilm exhibited weaker staining. The DAPI staining in the background of the biofilm was effectively eliminated from our analysis using the Imaris software. We encountered challenges with GFP-based detection as not all trophozoites expressed the same level of GFP, hindering accurate counting and mapping of trophozoites within the *B. subtilis* biofilm. Although DAPI staining is conventionally specific for nucleus staining, we employed it as an alternative method to detect trophozoites in our study. Supplementary Fig. 13 shows that all GFP-labeled trophozoites are also detected by DAPI. By utilizing DAPI staining, we were able to achieve more reliable results for trophozoite detection and localization within the biofilm. The fixed and stained biofilm was transferred to microscope slides and examined using an inverted confocal immunofluorescence microscope (Zeiss LSM700 meta laser scanning confocal imaging system, zoom 200). The Imaris software automatically detected and counted the trophozoites stained by DAPI. The number of trophozoites at various depths within the biofilm and the extent of biofilm destruction (loss of Mcherry signal) was assessed using the Imaris software.

### Scanning electron microscopy

The biofilms were developed over a mesh substrate. To preserve the intact biofilms, the mesh was carefully removed along with the biofilms. The samples were then fixed for 2–4 h at 4 °C in a solution containing 2% glutaraldehyde, 3% paraformaldehyde, 0.1 M sodium cacodylate (pH 7.4), and 5 mM CaCl_2_.

Following fixation, the samples underwent two 15-minute washes with double-distilled water to remove any residual fixative. Subsequently, a series of ethanol washes were performed to dehydrate the samples. Once dehydrated, the samples were left to dry overnight at room temperature.

Prior to examination, the samples were sputter-coated with a thin layer of gold-palladium. This coating process ensures optimal conductivity and sample stability during examination. Finally, the samples were examined using a scanning electron microscope (SEM) model XL30 equipped with a field emission gun.

### RNA extraction

Trophozoites were incubated with different forms of *B. subtilis* (planktonic or biofilm) or serum-free Diamond’s TYI S-33 medium without bacteria as a control. The trophozoites were incubated for 1 h at 37 °C in a 24-well plate that was covered with serum-free Diamond’s TYI S-33 agar.

After the incubation period, the trophozoites were harvested by gently tapping the glass tube, followed by centrifugation at 1900 rpm for 3 min. RNA extraction from *E. histolytica* was performed using the Monarch Total RNA Miniprep Kit (NEW ENGLAND BioLabs, Ornat, Nes Ziona, Israel). The extraction protocol was followed according to the manufacturer’s instructions, which emphasized the importance of avoiding mechanical disruption during the cell lysis step to ensure the extraction of *E. histolytica* RNA while minimizing extraction of *B. subtilis* biofilm RNA using the detergent-based lysis buffer.

### Library construction and sequencing

#### RNA QC

The quality assessment of RNA was performed using the TapeStation 4200 system from Agilent (Eldan Electronic Instrument, Petach-Tikva, Israel), along with the RNA ScreenTape & Reagents kit (cat no. 5067-5576). The obtained RINe values for all samples fell within the range of 8.9–9.9, signifying excellent RNA quality.

### Library constructions

Simultaneously, a total of 9 RNAseq libraries were constructed following the manufacturer’s protocol (NEBNext Ultra II Directional RNA Library Prep Kit for Illumina, cat no. E7760). Each library was prepared using 400 ng of total RNA as the starting material. To enrich for mRNA, a magnetic pull-down method was employed using the Magnetic Isolation Module (NEB, cat no. E7490). Subsequently, the concentration of each library was measured using Qubit (Invitrogen), while the size distribution was determined using the TapeStation 4200 with the High Sensitivity D1000 kit (cat no. 5067-5584). To ensure equal representation, all libraries were combined into a single tube with equal molarity. The RNAseq data was generated on an Illumina NextSeq2000 platform using P2 chemistry with 100 cycles (Read1-100; Index1-8; Index2-8) (Illumina, cat no. 20046811).

### NGS QC, alignment, and counting

Quality control analysis was performed using Fastqc (v0.11.8) to evaluate the sequencing data. Following this, reads were subjected to trimming for adapter sequences, removal of low-quality bases from the 3’ end, and a minimum length threshold of 20 using CUTADAPT (v1.10). The resulting 100 bp single reads were aligned to the reference genome of amoeba *E. histolytica* (strain HM1IMSS). The reference genome can be accessed at the following URL: https://amoebadb.org/common/downloads/Current_Release/EhistolyticaHM1IMSS/fasta/data/. In addition, an annotation file providing information about the genome can be found at: https://amoebadb.org/common/downloads/Current_Release/EhistolyticaHM1IMSS/gff/data/.

For the alignment process, Tophat2 version 2.1.0 (utilizing Bowtie2 version 2.2.6) was employed. Subsequently, the number of reads mapped to each gene was quantified using Htseq-count (v0.11.2).

### Descriptive analysis

A statistical analysis was pre-formed using DESeq2 R package (version 1.28.1) (Genome Biology 2014 15:550). The number of reads per gene was extracted into merged_counts.csv and normalized_counts.csv files for raw counts and normalized counts, respectively. The similarity between samples was evaluated within DESeq2 package using correlation matrix, shown in heatmap plot.

### Differential expression analysis and GO terms

Using the DESeq2 statistical model, we identified differentially expressed genes (DEGs) in each comparison. DEGs were determined based on an adjusted *p*-value threshold of <0.05 (FDR), considering all genes that passed DESeq’s independent filtering thresholds.

Volcano plots were generated to visualize the DEGs. In these plots, upregulated genes were depicted in light blue, while downregulated genes were represented in red, relative to each specific comparison. Notably, specific DEGs belonging to selected computed Gene Ontology (GO) function terms were marked using triangles in the volcano plots, whereas all other genes were represented by circles.

The selected computed GO function terms were associated with two biological functions of interest: “Cysteine Proteases,” and “Dehydrogenase.” More detailed information regarding these specific genes and their functional assignments can be found in supplementary Table 2. The GO function assignments were generated by VEuPathDB using InterPro-to-GO and obtained from AmoebaDB.

### Availability of data

RNA-Seq data are available at the Gene Expression Omnibus (http://www.ncbi.nlm.nih.gov/geo accessed on “date”) under the accession number GSE233645.

### Quantification of trophozoites deepness in *B. subtilis* biofilm

The microscopy images were analyzed using Imaris software. Each trophozoite in the biofilm was identified based on its DAPI staining. Once the trophozoites were identified, Imaris generated an Excel file containing the depth of each trophozoite (i.e., their distance from the biofilm surface) in each biofilm. The analysis involved comparing two conditions: WT (wild type) and WT treated with E64D.

### Role of EhCPs in the degradation of *B. subtilis* biofilm

To investigate the impact of CPs on the ability of *E. histolytica* trophozoites to degrade *B. subtilis* biofilm CPs inhibitor E64D was used (10 µM). This concentration has been previously established as effective for inhibiting CPs^86^. Trophozoites (1 × 10^6^) were incubated with E64D for 24 h at 37 °C. The viability of the trophozoites was assessed using the eosin exclusion assay^87^. In each experimental condition, 1 × 10^6^ living trophozoites were incubated with the biofilm for 3 h at 37 °C. As a negative control, trophozoites fixed with 4% paraformaldehyde were used. Subsequently, the biofilms were washed once with serum-free Diamond’s TYI S-33 medium to remove the trophozoites. The measurement of biofilm degradation was carried out using the method described in biofilm degradation assay, allowing us to assess the extent of degradation under each condition.

### Silencing of EhCP5 (EH_168240) gene, transfection

To generate the siEhCP5 silencing vector for suppressing EhCP5 expression, we performed the following steps. Firstly, EhCP5 (EH_168240) was amplified from *E. histolytica*’s genomic DNA using specific primers (5’EhCP5 and 3’EhCP5) as listed in supplementary Table 3. The resulting PCR product was then cloned into the pGEM-T Easy vector system (Promega, WI, USA). Subsequently, the cloned plasmid was subjected to digestion using BglII and XhoI restriction enzymes. Next, the digested DNA insert containing EhCP5 was subcloned into the *E. histolytica* pEhEx-04-trigger silencing vector (a kind gift of Dr. Tomoyoshi Nozaki at the University of Tokyo, Japan). The insertion of the EhCP5 insert into the pEhEx-04-trigger vector resulted in the generation of the siEhCP5 vector. To ensure the presence of the correct EH_168240 gene sequence, the resulting plasmid was sent for sequencing analysis. The transfection of *E. histolytica* trophozoites was carried out by utilizing lipofectAMINE-silencing plasmid DNA complexes, which were prepared in OPTI-MEM I medium (Life Technologies, Rhenium, Modi’in, Israel). The transfected trophozoites were then selected in TYI-S-33 medium with the addition of 3 μg/ml G418. Following selection, the trophozoites were maintained in the presence of 6 μg/ml G418, as described previously^88^. Following selection, the trophozoites were maintained in the presence of 6 μg/ml G418, as described previously^88^.

### CP activity assay

CP activity was determined in total lysates of *E. histolytica* trophozoites (1 × 10^6^) using a lysis buffer containing 1% Nonidet P-40 (NP-40 in Deuterium-depleted water (DDW)). The measurement of CP activity was performed following a previously described protocol^89^. The enzymatic activity was quantified based on the digestion of Z-Arg-Arg-pNA substrate (BACHEM), and one unit of CP activity was defined as the amount of enzyme that digests one micromole of Z-Arg-Arg-pNA per minute per milligram of protein. CP activity was determined in total lysates of *E. histolytica* trophozoites (1 × 10^6^) using a lysis buffer containing 1% Nonidet P-40 (NP-40 in DDW). The measurement of CP activity was performed following a previously described protocol^89^. The enzymatic activity was quantified based on the digestion of Z-Arg-Arg-pNA substrate (BACHEM), and one unit of CP activity was defined as the amount of enzyme that digests one micromole of Z-Arg-Arg-pNA per minute per milligram of protein.

### Determination of the response of treated and untreated biofilms to antibiotics

The sensitivity of treated and untreated biofilms to antibiotics was tested as described previously, with mild modifications^38^. Biofilms of indicated strains were grown on solid MSgg medium as described, with or without the presence of the extract or extract with E64D (25% total value, 2 μg/ml). After 3 days, the colonies were cut in half with a razor blade. One-half of the colony was exposed to 500 μl chemical stress [50% (v/v) 0.05% (v/v) sodium hypochlorite (Bio-Lab Chemicals) or 600 ng/ml Ampicillin. The second half of the colony was incubated in PBS. After 20 min (sodium hypochlorite) or 4 h (ampicillin) of incubation, biofilms were centrifuged (5 min at 14,000 r.p.m), the supernatant was removed, and biofilms were resuspended in 500 μl PBS and mildly sonicated (amplitude 20%, pulse 3 × 5 s). The number of CFU was determined by plating serial dilutions on LB plates and counting colonies after incubation at 30°C overnight.

### Determination of the biding mechanism of trophozoites on biofilms

The aim of this experimental setup was to gain insights into the nature of the receptor involved in the interaction between *E. histolytica* trophozoites and *B. subtilis* or *E. coli* GFP-expressing biofilms. To achieve this, trophozoites (1 × 10^6^) were incubated with the biofilm for 3 h for *B. subtilis* and 1 h for *E. coli* at 37 °C. In order to investigate potential receptors, various competitors including galactose (2%), mannose (1%), asialofetium (0.05%), gelatin (1%), TasA (0.01%), planktonic form (1 × 10^9^), and sonicated biofilm (biofilm from a single well was sonicated for 3 cycles of 15 s on/off at medium intensity on a bio-ruptor UCD 200) of *B. subtilis* were added to each biofilm. To determine biofilm degradation, the remaining GFP signal intensity was measured as an indication of biofilm integrity after the action of *E. histolytica* trophozoites as described in biofilm degradation assay.

### TasA purification

Protein was expressed and purified as previously described^90^. TasA was purified using the pDFR6 (pET22b-*tasA*) and *E. coli* BL21(DE3) cells were freshly transformed with the plasmid. Colonies were selected from the plates and resuspended in 10 ml of LB with 100 µg/ml of ampicillin and incubated overnight at 37 °C with shaking. This pre-inoculum was then used to inoculate 500 ml of LB + ampicillin, and the culture was incubated at 37 °C until an OD600 of 0.7–0.8 was reached. Next, the culture was induced with 1-mM isopropyl β-D-1-thiogalactopyranoside (IPTG) and incubated O/N at 30 °C with shaking to induce the formation of inclusion bodies. After that, cells were harvested by centrifugation (5000 × *g*, 15 min, 4 °C) resuspended in buffer A (Tris 50 mM, 150 mM NaCl, pH8), and then centrifuged again. These pellets were stored frozen at −80 °C until used. After thawing, cells were resuspended in buffer A, and broke down by sonication on ice using a Branson 450 digital sonifier (3 × 45 s, 60% amplitude). After sonication, the lysates were centrifuged (15,000 × *g*, 60 min, 4 °C) and the supernatant was discarded, as proteins were mainly expressed in inclusion bodies. The proteinaceous pellet was resuspended in buffer A supplemented with 2% Triton X-100, incubated at 37 °C with shaking for 20 min, to further eliminate any remaining cell debris, and centrifuged (15,000 × *g*, 10 min, 4 °C). The pellet was then extensively washed with buffer A (37 °C, 2 h), centrifuged (15,000 × *g* for 10 min, 4 °C), resuspended in denaturing buffer (Tris 50 mM NaCl 500 mM, 6 M GuHCl), and incubated at 60 °C overnight to completely solubilize the inclusion bodies. Lysates were clarified via sonication on ice (3 × 45 s, 60% amplitude) and centrifugation (15,000 × *g*, 1 h, 16 °C) and were then passed through a 0.45-µm filter prior to affinity chromatography. Proteins were purified using an AKTA Start FPLC system (GE Healthcare). The lysates were loaded into a HisTrap HP 5 ml column (GE Healthcare) previously equilibrated with binding buffer (50 mM Tris, 0.5 M NaCl, 20 mM imidazole, 8 M urea, pH 8). Protein was eluted from the column with elution buffer (50 mM Tris, 0.5 M NaCl, 500 mM imidazole, 8 M urea, pH 8). After the affinity chromatography step, buffer was exchanged to 1% acetic acid pH 3, 0.02% sodium azide by using a HiPrep 26/10 desalting column (GE Healthcare). This ensured that the proteins were maintained in their monomeric form. The purified proteins were stored under these conditions at 4 °C (maximum 1 month) until further use.

### TasA degradation by trophozoites lysate

Trophozoites were lysed by incubating them with NP-40 buffer (1% NP40 in DDW) for 15 min on ice. Subsequently, TasA (2 µg) was added to the trophozoites lysate (20 µg), or with a trophozoites lysate treated with E64D (20 µg), and the mixture was incubated for 3 h at 37 °C in a final volume of 20 µl of NP-40 buffer, supplemented with DTT (5 mM). The samples were analyzed using 12% SDS-PAGE, followed by Coomassie staining. As controls, TasA and trophozoites lysate were incubated separately without any additional treatments.

### Detection of oxidized proteins in trophozoites

The level of oxidized protein was measured with the Oxyblot kit (Protein Carbonyl Assay Kit, Abcam) according to the manufacturer protocol. Trophozoites (1 × 10^6^) were incubated for 1 h at 37 °C with *B. subtilis* planktonic form, *B. subtilis* biofilm form or with serum-free Diamond’s TYI S-33 medium without bacteria (as control) in 24-well plates cover with serum-free Diamond’s TYI S-33 agar. Then, the cells were exposed to H_2_O_2_ (2.5 mM, for 30 min at 37 °C). Trophozoites were then lysed with Nonidet P-40 (NP-40 1% in DDW) for 15 min on ice. Equal protein concentrations (20 μg) were proceeded with the oxyblot, protein oxidation detection kit.

### Detection of reactive oxygen species in trophozoites in contact with *B. subtilis* biofilm and exposed to H_2_O_2_

To assess the ROS levels in trophozoites, the trophozoites were incubated with *B. subtilis* biofilm for 3 h at 37 °C. Subsequently, H_2_O_2_ (0.5 mM) was added on the top of biofilms and incubated for 10 min at 37 °C. Biofilm were wash one time with serum-free Diamond’s TYI S-33 medium to remove the H_2_O_2._ A fluorescent probe, 2’,7’-dichlorodihydrofluorescein diacetate (H2DCFDA, 10 µM), was added to each well and incubated for 30 min at 37 °C in the dark, within TYI medium. The biofilm were then fixed with paraformaldehyde (PFA, 4%) for 30 min at room temperature (in the dark). Trophozoites were subsequently stained with DAPI (20 µg/ml) for 1 h at 4 °C in the dark. Finally, the samples containing biofilm were examined using an inverted confocal immunofluorescence microscope (Zeiss LSM700 meta laser scanning confocal imaging system, zoom 200). The level of ROS in trophozoites cells was quantified using Imaris software. The trophozoites and biofilm were distinguished in confocal microscopy images by their fluorescence (red for the biofilm and green for the trophozoites). The fluorescence of the H2DCFDA probe (green) was then used to quantify the oxidation level in each trophozoites cell within the biofilm. After Imaris isolated each trophozoites having H2DCFDA probe signal, the software could give the intensity of each cell and its deepness in the biofilm. For additional analysis, we opted to categorize the trophozoites into two distinct groups: trophozoites residing on the biofilm’s surface (above 15 µm) and trophozoites positioned deeper within the biofilm (below 15 µm).

### Cytopathic assay in presence of *B. subtilis* biofilm

In this modified cytopathic assay, a pre-formed biofilm of *B. subtilis* was placed on top of a monolayer of Caco-2 cells in a well containing 2 ml of serum-free TYI-S-33 medium. Subsequently, 3 × 10^5^ *E. histolytica* trophozoites were added to the top of the biofilm. Following a 60-minute incubation period, we quantified the destruction of the Caco-2 cell monolayer induced by the parasite, as described in a previous study^91^. To establish appropriate controls, we incubated the parasite with the Caco-2 cell monolayer in the absence of bacteria and with a suspension of 3 × 10^8^ planktonic *B. subtilis* cells. Furthermore, to quantify the extent of damage inflicted by *E. histolytica* trophozoites on Caco-2 cells, we employed methylene blue staining (0.1% in 0.1 M borate buffer, pH 8.7) to visualize and count the remaining attached Caco-2 cells, following established procedures^92^.

### Biofilm destruction quantification using crystal violet assay

For biofilm growth, 1 μl of LGG starter culture was diluted (1:100) in 100 µl TSB, in 96-well polystyrene plates and incubated for overnight in 37 °C. Then planktonic cells were removed by pipetting, and wells were incubated with the indicated solutions for 2 h. The attached biomass was washed with DDW. The adherent cells were stained with 0.05% crystal violet stain for 30 min. The stain was removed, and the wells were washed with DDW. 100% ethanol was added to the wells for 15 min. Crystal violet intensity was determined by a spectrophotometer (OD 570 nm).

### Biofilm/planktonic ratio assay

For biofilm growth, 1 µl of indicated strain starter culture was diluted (1:100) in 100 µl TSB, in 96-well polystyrene plates and incubated for overnight in 37 °C. Then, treatment solutions were added to each well as indicated in the figure legends for 2 h. The planktonic cells were removed and analyzed for CFU formation. The adherent cells resuspended in PBS (100 µl) and analyzed for CFU formation. The samples were serially diluted x10 into 96-well plates and 20 µl from each sample was plated on solid LB agar (1.5% agar) using a multichannel pipette with the dot-spot technique. CFU enumeration was carried out following overnight incubation at 37 °C. The ratio calculated was adherent cells CFU/Planktonic cells CFU.

### CPs secretion by *E. histolytica* incubated with TasA

Trophozoites (10^6^) were incubated with different concentrations of TasA (2 μg, 5 μg, and 10 μg) for 3 h at 37 °C in 500 µl of TYI-S-33 medium without serum which was chosen as secretion medium. After incubation, trophozoites were centrifuged (1900 rpm for 3 min at RT), and the secretion product was isolated and run on 12% SDS-PAGE or on a gelatin gel (1% gelatin), followed by staining with Coomassie^93^. As a control, TasA and the secretion product were incubated alone. Furthermore, the cytoplasmic alcohol dehydrogenase (EhADH) activity was measured as an additional marker to evaluate the integrity of the trophozoites in the secretion medium^59^.

### CsgA purification

*E coli* CsgA strain NEB 3016 slyD-/pET11d + Sec- csgA His6 was grown overnight with ampicillin^94^. At OD600 = 0.85–0.9, 1 M of IPTG (final conc. is 0.5 mM) was added to induce CsgA expression. Cultures were pelleted and stored at −80 °C. Pellet was dissolved in 8 M Guanidine and incubated for 1 h at room temperature. Solution was centrifuged and the supernatant was sonicated at room temperature. Nickel affinity beads were added to sonicated supernatant and incubated for 1 h at room temperature. 50 mM KPi pH 7.3 with 12.5 mM imidazole was used to wash the affinity column followed with elution of *E. coli* CsgA with 50 mM KPi pH 7.3 with 125 mM imidazole. The eluent was then desalted and buffer exchanged into 50 mM KPi pH 7.3 to remove imidazole.

### The effect of *E. histolytica* trophozoites lysate on CsgA

The trophozoites were lysed with NP-40 (1% in DDW) for 15 min on ice. Subsequently, CsgA (2 µg) was incubated with the trophozoites lysate (20 µg), as well as the trophozoites lysate treated with E64D (20 µg), for 3 h at 37°C in a final volume of 20 µl containing DTT (1 M). The different conditions were analyzed using 15% SDS-PAGE^95^ gel, followed by Coomassie staining for protein visualization. As controls, CsgA and trophozoites lysate were separately incubated without any additional treatments.

### Statistical analysis

Statistical analyses were performed with GraphPad Prism 9.0 (GraphPad 234 Software, Inc., San Diego, CA). The test used for pair-wise experiments was an unpaid T test. The *P*-value were, *p < 0.05, **p < 0.01, ***p < 0.001, and ****p < 0.0001. Otherwise, the test used was a multiple comparison tests (Anova) as indicated in the legends of each figure. Statistical tests are mentioned in the indicated legends of the figures.

### Reporting summary

Further information on research design is available in the Nature Research Reporting Summary linked to this article.

### Supplementary information

Supplementary information

supplementary table 1

Reporting Summary

## Data Availability

All required data for the main and supplementary figures are provided with the manuscript and the supplementary information file.
