# Supplementary material for: Digestive exophagy of biofilms by intestinal amoeba and its impact on stress tolerance and cytotoxicity
Source: NPJ Biofilms Microbiomes. 2023 Oct 9;9:77. doi: 10.1038/s41522-023-00444-x (PMC10562373; doi:10.1038/s41522-023-00444-x)
Supplement: Supplementary file 1 — Supplementary information [file 41522_2023_444_MOESM1_ESM.pdf]

Digestive exophagy of biofilms by intestinal amoeba and its impact on stress tolerance and cytotoxicity

Eva Zanditenas[1], Meirav Trebicz-Geffen[1], Divya Kolli[2#], Laura Domínguez-García[3#], Einan Farhi[4], Liat Linde[4], Diego Romero[3] , Matthew Chapman[2], Ilana Kolodkin-Gal[5,6]* and Serge Ankri[1] *

[1]Department of Molecular Microbiology, Ruth and Bruce Rappaport Faculty of Medicine, Technion, Haifa, Israel

[2]Department of Molecular, Cellular, and Developmental Biology, University of Michigan, Ann Arbor, USA

[3]Departamento de Microbiología, Instituto de Hortofruticultura Subtropical y Mediterránea 'La Mayora', Universidad de Málaga-Consejo Superior de Investigaciones Científicas (IHSM-UMA-CSIC), Universidad de Málaga, Málaga, Spain

[4]Technion Genomics Center, Technion- Israel Institute of Technology

[5]Department of Plant Pathology and Microbiology, the Robert H. Smith Faculty of Agriculture, Food & Environment, The Hebrew University of Jerusalem, Rehovot, Israel

[6]Scojen Institute for Synthetic Biology, Reichman University, Herzliya, IL

#Contributed equally

*Corresponding authors: ilana.kolodkin@runi.ac.il; sankri@technion.ac.il

Supplementary Information

Supplementary Figures (1-14)

Supplementary Tables (1-3)

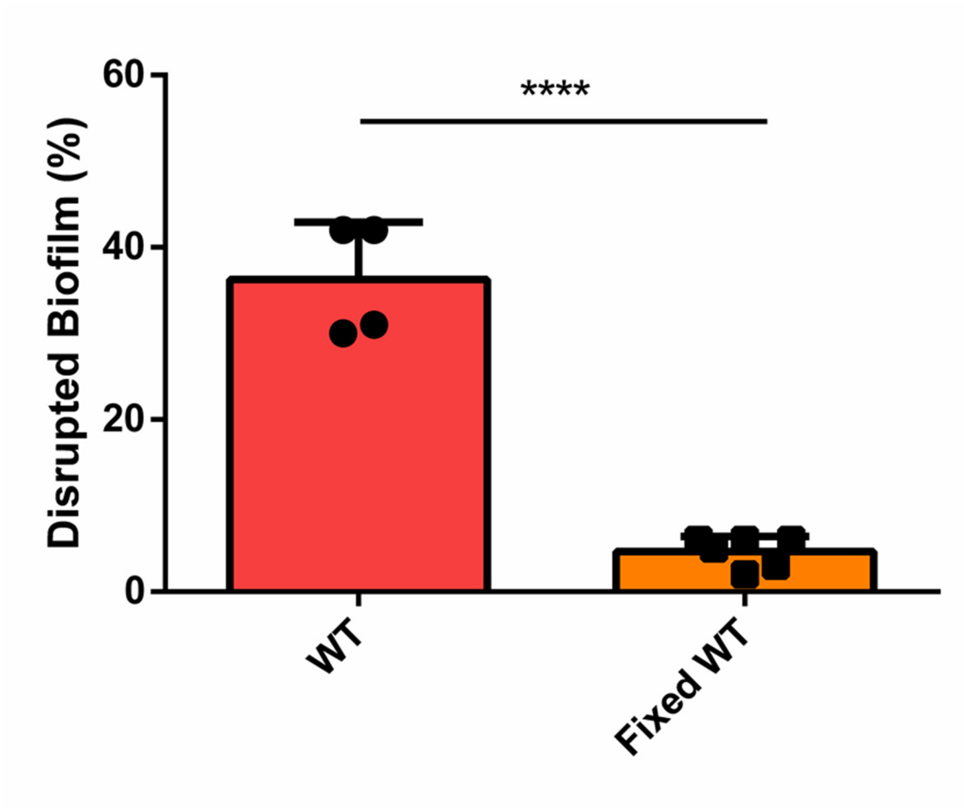

Supplementary Figure 1: Fixed trophozoites were used as negative control for *B. subtilis* biofilms destruction by *E. histolytica* trophozoites.

GFP intensity of each biofilm was measured using ImageJ, with fixed trophozoites (treated with paraformaldehyde 4%) incubated with *B. subtilis* biofilm (WT), Fixed trophozoites were treated with 4% paraformaldehyde and incubated with *B. subtilis* biofilm (Fixed WT), resulting in no degradation of the biofilm. Statistical analysis was performed using an unpaired T-test, **** indicates p-value less than 0.0001. Data represent the average results from three biological replicates.

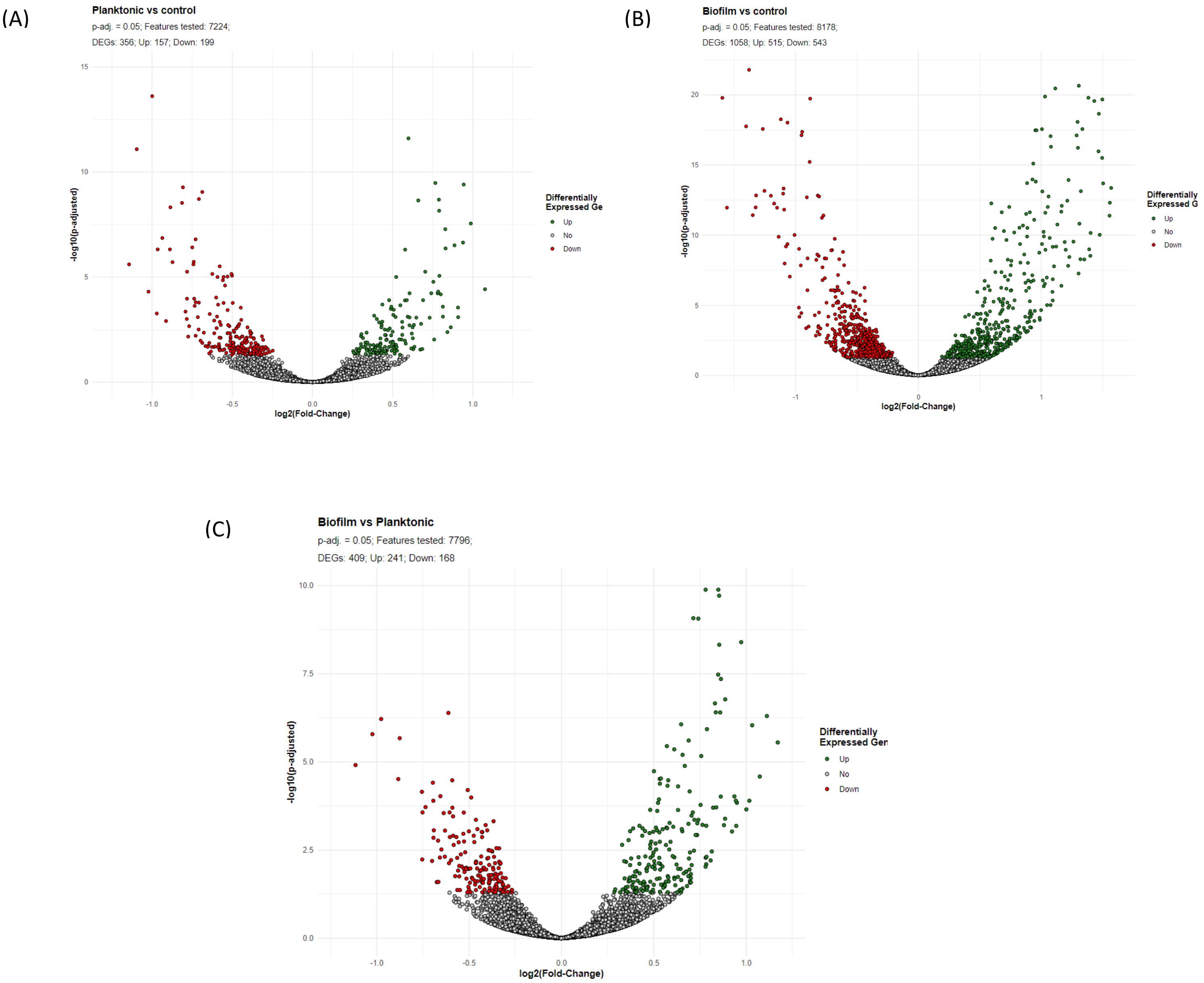

Supplementary Figure 2: Volcano plot analysis of differentially expressed genes in *E. histolytica*.

The volcano plots depict the gene expression profiles for different experimental conditions, with significant downregulated genes represented by red dots and significant upregulated genes represented by green dots. Light gray dots indicate genes that do not exhibit significant changes in expression. The gene expression analyses were performed for the following experimental comparisons:

(A) Gene expression in *E. histolytica* trophozoites incubated with planktonic *B. subtilis* compared to *E. histolytica* trophozoites alone (control group).

(B) Gene expression in *E. histolytica* trophozoites incubated with *B. subtilis* biofilm compared to *E. histolytica* trophozoites alone (control group).

(C) Gene expression in *E. histolytica* trophozoites incubated with *B. subtilis* biofilms compared to the planktonic *B. subtilis* cells.

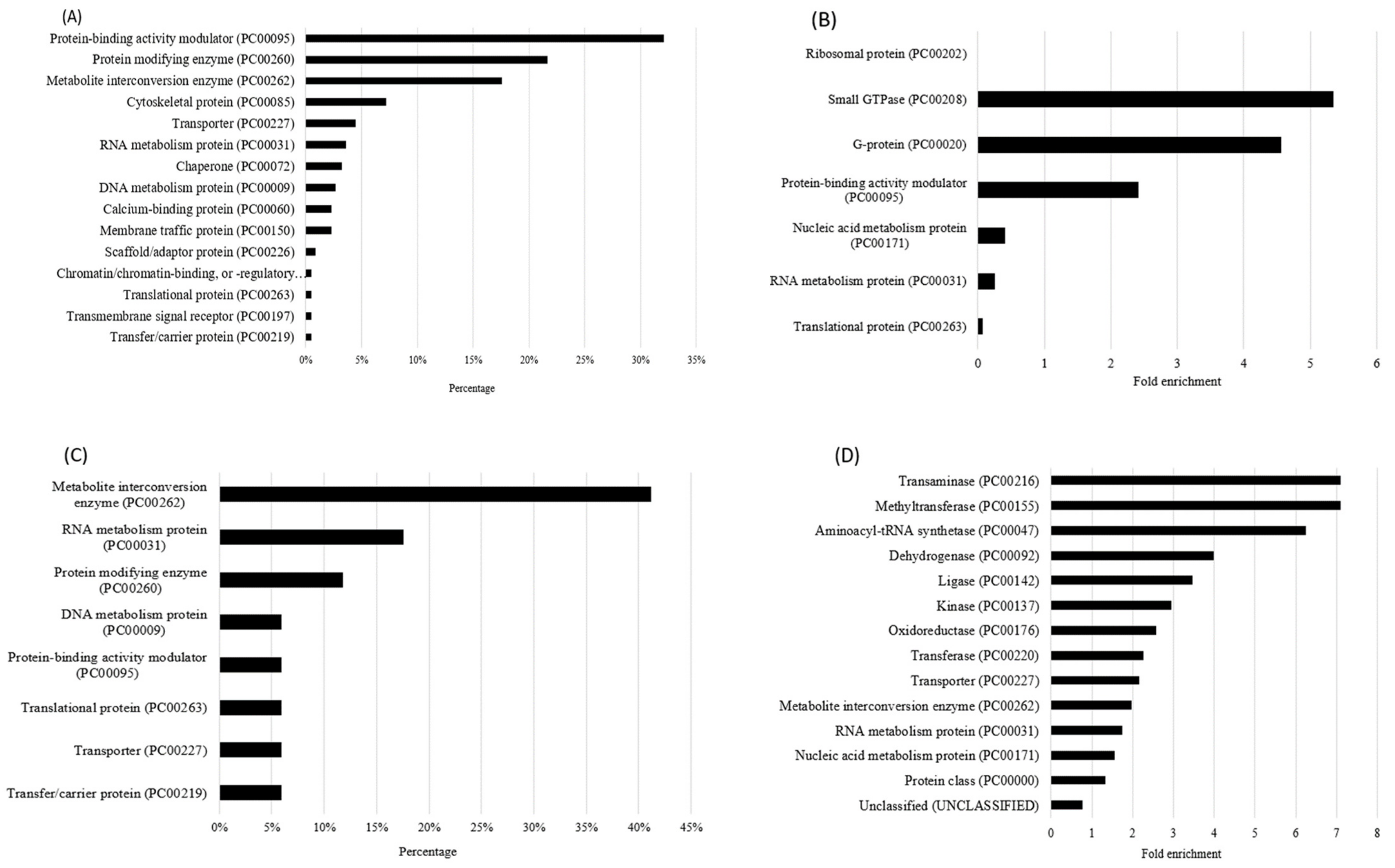

Supplementary Figure 3: PANTHER sequence classification and statistical overrepresentation tests for upregulated and downregulated genes in trophozoites incubated with biofilm form vs. control trophozoites.

(A) PANTHER sequence classification of upregulated genes (B) PANTHER statistical overrepresentation test of upregulated genes (C) PANTHER sequence classification of downregulated genes (D) PANTHER statistical overrepresentation test of downregulated genes.

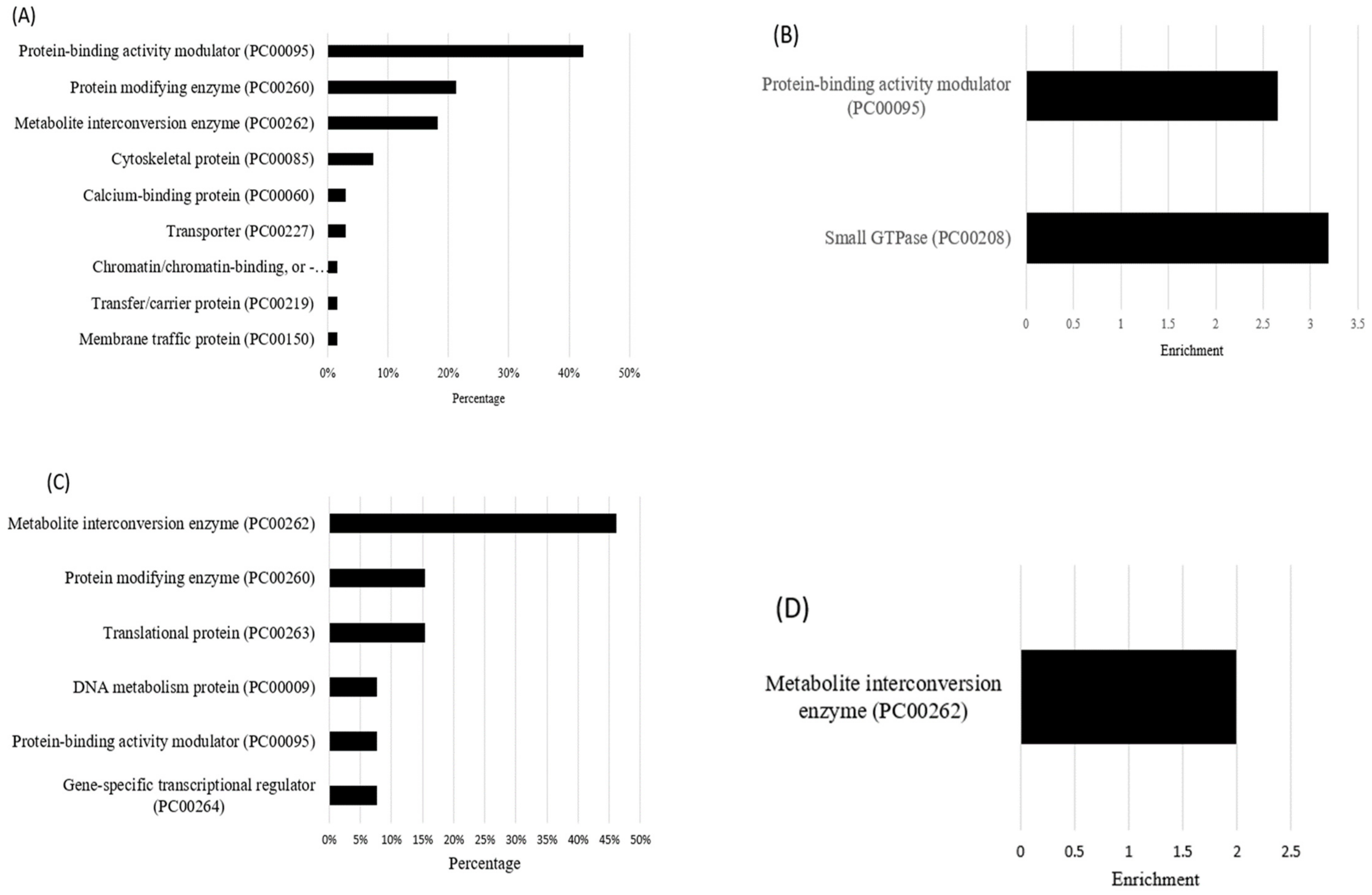

Supplementary Figure 4: PANTHER sequence classification and statistical overrepresentation tests for upregulated and downregulated genes in trophozoites incubated with planktonic form of *B. subtilis* VS control trophozoites.

(A) PANTHER sequence classification of upregulated genes. (B) PANTHER statistical overrepresentation test of upregulated genes. (C) PANTHER sequence classification of downregulated genes. (D) PANTHER statistical overrepresentation test of downregulated genes.

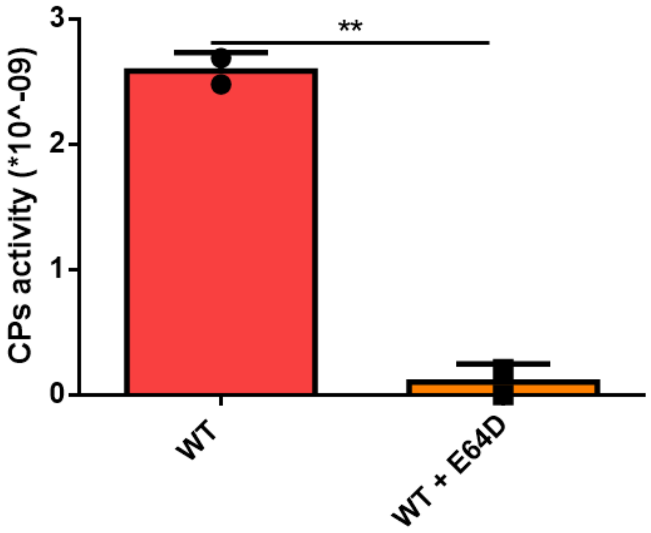

Supplementary Figure 5: Inhibition of EhCP activity in *E. histolytica* trophozoites by E64D. To assess the inhibitory effect of E64D on EhCP activity, control trophozoites (WT) and trophozoites treated with E64D (10 µM for 24 hours) (WT + E64D) were analyzed. CP activity was measured by monitoring the cleavage of the Z-Arg-Arg-pNA substrate. One unit of CP activity was defined as the amount of enzyme capable of digesting one micromole of Z-Arg-Arg-pNA per minute per milligram of protein.

Significant differences in CP activity were determined using an unpaired T-test (p-value ** is less than 0.001). The data in this figure were obtained from two independent biological replicates.

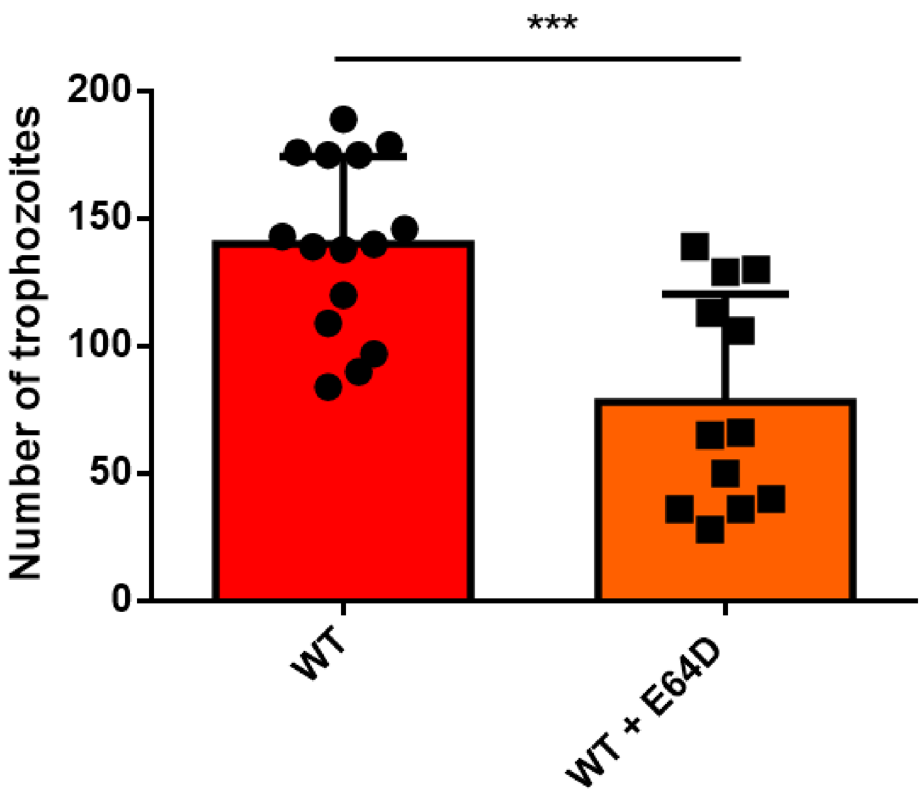

Supplementary Figure 6: Viability of trophozoites after treatment with E64D.

The viability of trophozoites was evaluated following treatment with E64D. Trophozoites from the control group (WT) and those cultivated with E64D (10 μM for 24 hours at 37ºC) (WT + E64D) were subjected to viability assessment using the eosin assay. Statistical analysis was performed using an unpaired T-test, and a p-value of *** is less than 0.001) indicates a significant difference between the groups.

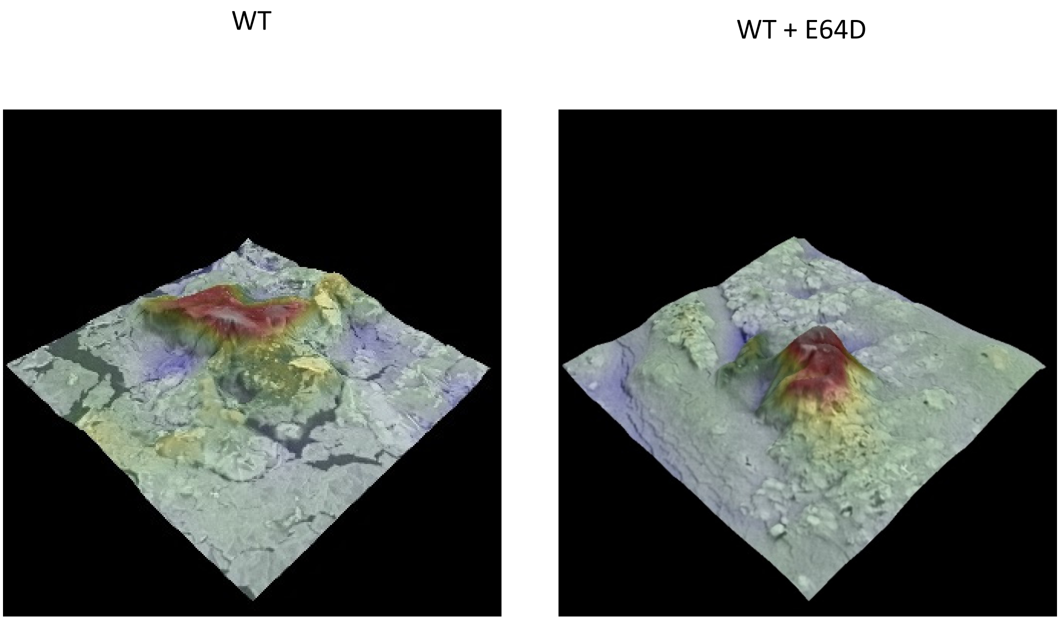

Supplementary Figure 7: A heat map depicting the thickness of biofilms incubated with *E. histolytica* trophozoites, treated with and without E64D.

Gradients are from low-blue, to high- red. The heat map was analyzed by scanning electron microscopy (Figs 2D and E) demonstrates the parasite footprint as well as areas of clearance in biofilms treated with trophozoites in the absence of E64D but not its presence. Data are representative of three independent experiments.

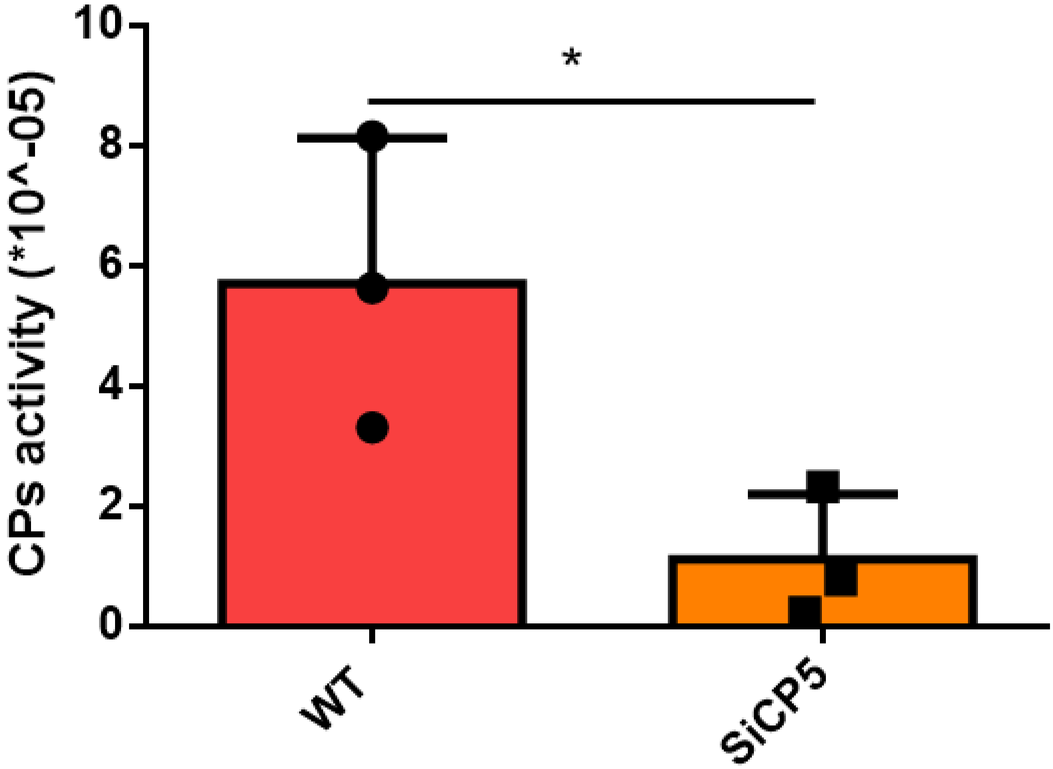

Supplementary Figure 8: CP activity in *E. histolytica* trophozoites VS CP5-Silenced trophozoites.

CP activities were assessed in *E. histolytica* trophozoites (WT) and trophozoites with CP5 gene silenced (SiCP5). The activity of CPs was measured by monitoring the cleavage of the Z-Arg-Arg-pNA substrate. One unit of CP activity was defined as the amount of enzyme capable of digesting one micromole of Z-Arg-Arg-pNA per minute per milligram of protein. Statistical analysis was conducted using a T-test, and a p-value of * is less than 0.05. The data presented in this figure represent the average results obtained from three independent biological replicates.

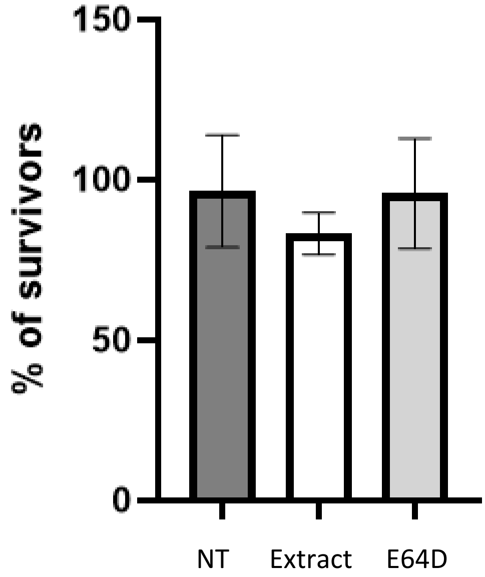

Supplementary Figure 9: The neutral effect of *E. hystolytica* extract on biofilm cells' viability. Results represent an average and standard deviation of five independent repeats*. B. subtilis* NCIB3610 and its indicated mutants were grown on defined MSgg agar (Bucher et al., 2016) at 30°C for 48 hours. Then, the colonies were cut in half with a razor blade. To determine the susceptibility to ampicillin or sodium hypochlorite within a biofilm, cell-number percentage of CFU without or with chemical stress was compared. Then, biofilms were split into two equal halves and re- suspended in 100µl PBS/Extract/Extract+E64D for 4 hours. Cells were pelleted and resuspended either in PBS 500 µl and analyzed for CFU formation. The samples were serially diluted x10 into 96 well plates and 20 µL from each sample was plated on solid LB agar (1.5 % agar) using a multichannel pipette with the dot-spot technique. CFU enumeration was carried out following overnight incubation at 37°C.

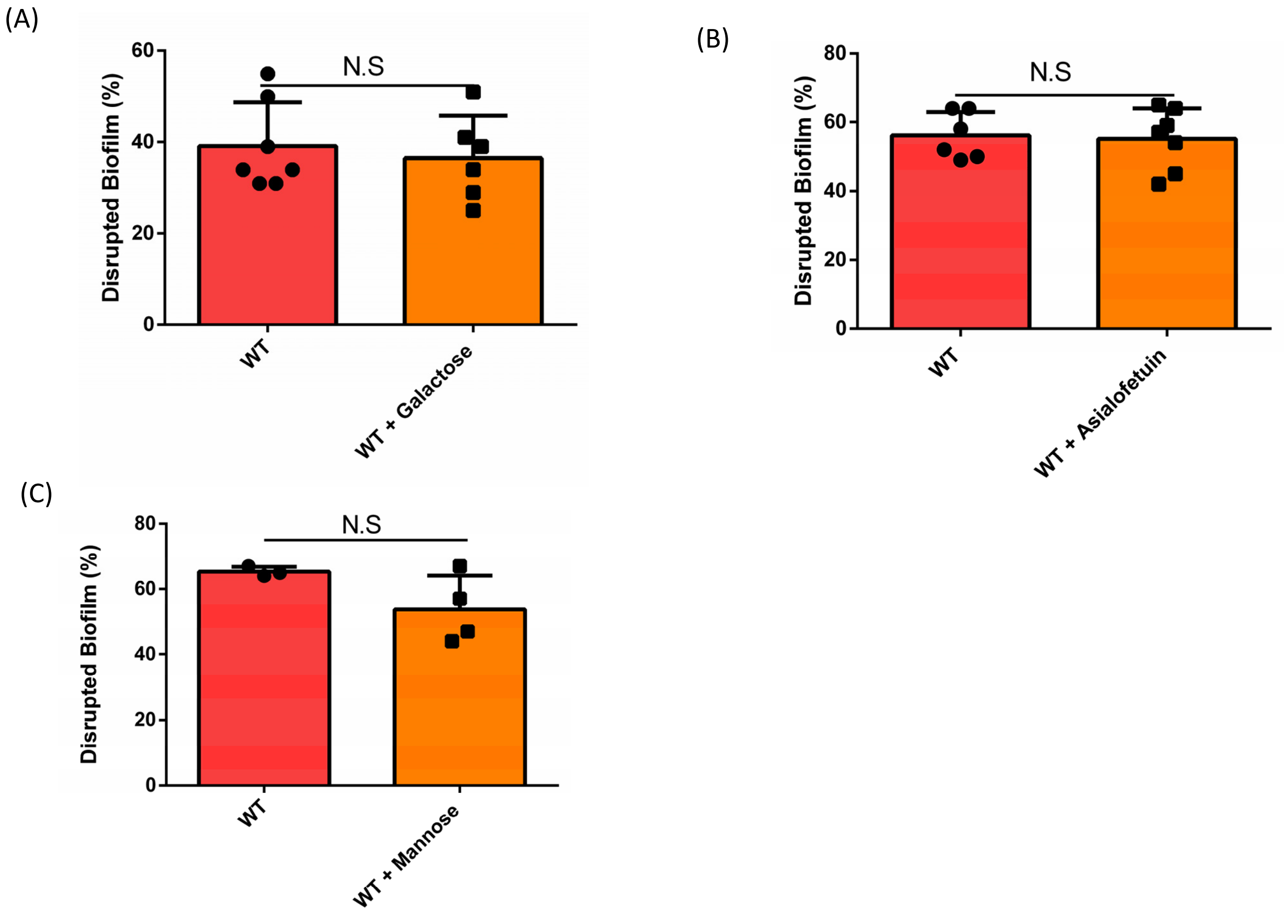

Supplementary Figure 10: Effect of galactose, asialofetuin and mannose on the degradation of *B. subtilis* biofilm by *E. histolytica* trophozoites.

The quantification of *B. subtilis* biofilm degradation by *E. histolytica* trophozoites (WT), along with trophozoites incubated with (A) galactose (2%) (WT+Galactose), (B) asialofetuin (0.05%) (WT+Asialofetuin) and (C) mannose (2%) (WT+Mannose) was performed following the methodology outlined in the Materials and Methods section. Statistical analysis was performed using an unpaired T-test, the data are not significant (N.S). Data represent averages of results from three biological replicates.

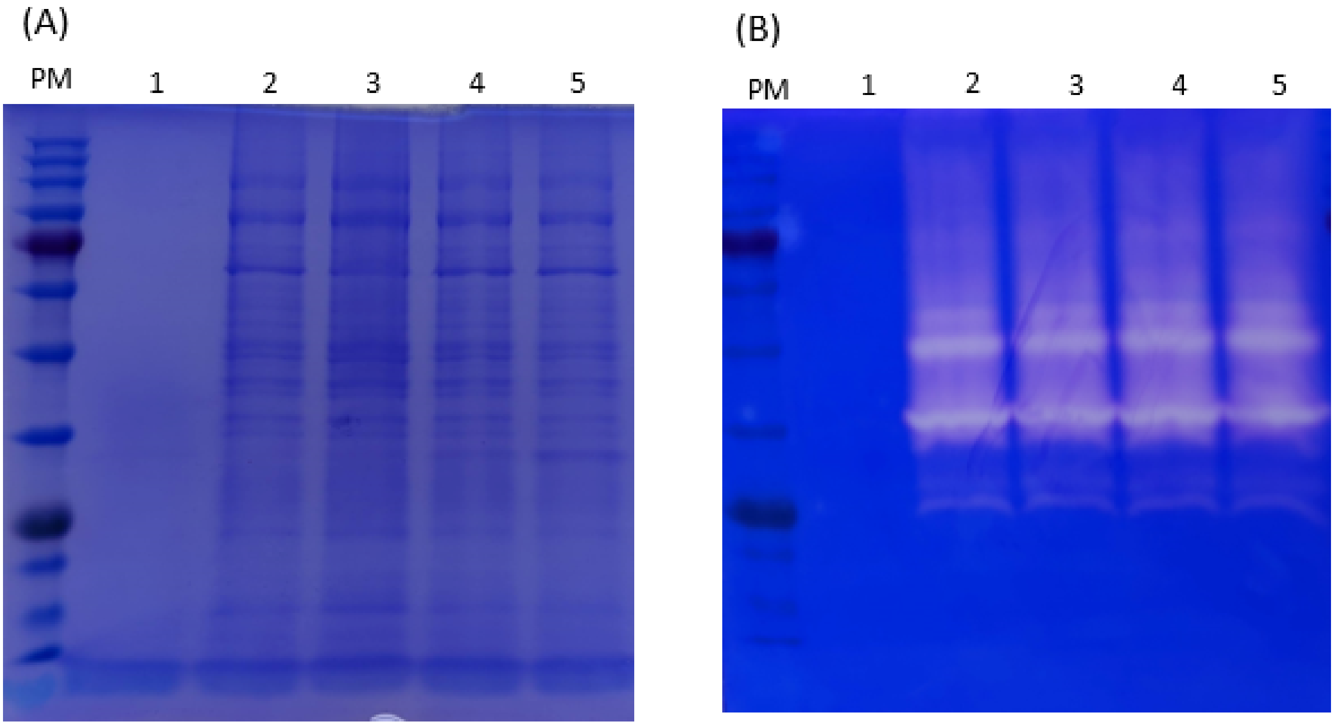

Supplementary Figure 11: Effect of TasA on the Secretion of EhCPs by *E. histolytica* Trophozoites.

E. histolytica trophozoites were incubated with different concentrations of TasA in 500 µl of TYI secretion medium at 37°C for three hours. Subsequently, the proteins present in the secretion products were separated using (A) 12% SDS-PAGE or (B) gelatin substrate gel, followed by staining with Coomassie Blue. These gels are representative of three repeats.

Legend: (PM) Protein marker, (1) TasA (2 µg), (2) Secretion product (SP) of trophozoites incubated without TasA, (3) SP of trophozoites incubated with TasA (2 µg), (4) SP of trophozoites incubated with TasA (5 µg), (5) SP of trophozoites incubated with TasA (10 µg).

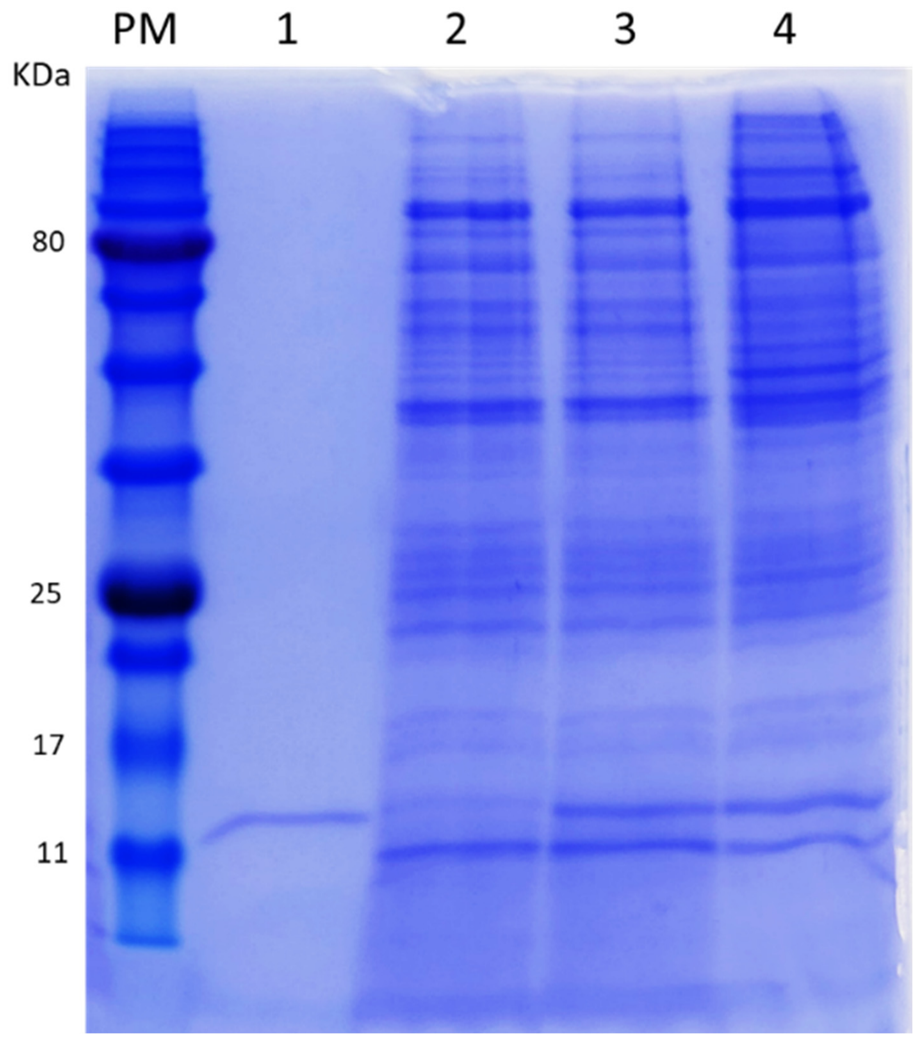

Supplementary Figure 12: The effect of *E. histolytica* total lysate on CsgA.

CsgA was incubated with lysate of trophozoites treated or not with E64D for 180 minutes at 37°C and its degradation by proteases present in *E. histolytica* lysate was analyzed by SDS-PAGE and Coomassie staining. This gel is representative of three repeats.

(1) CsgA (2 µg), (2) Control trophozoites lysate (20 µg of proteins) (WT), (3) CsgA (2 µg) + WT lysate (20 µg of proteins), (4) CsgA (2 µg) + lysate of E64D (10 µg) treated trophozoites (20 µg of proteins).

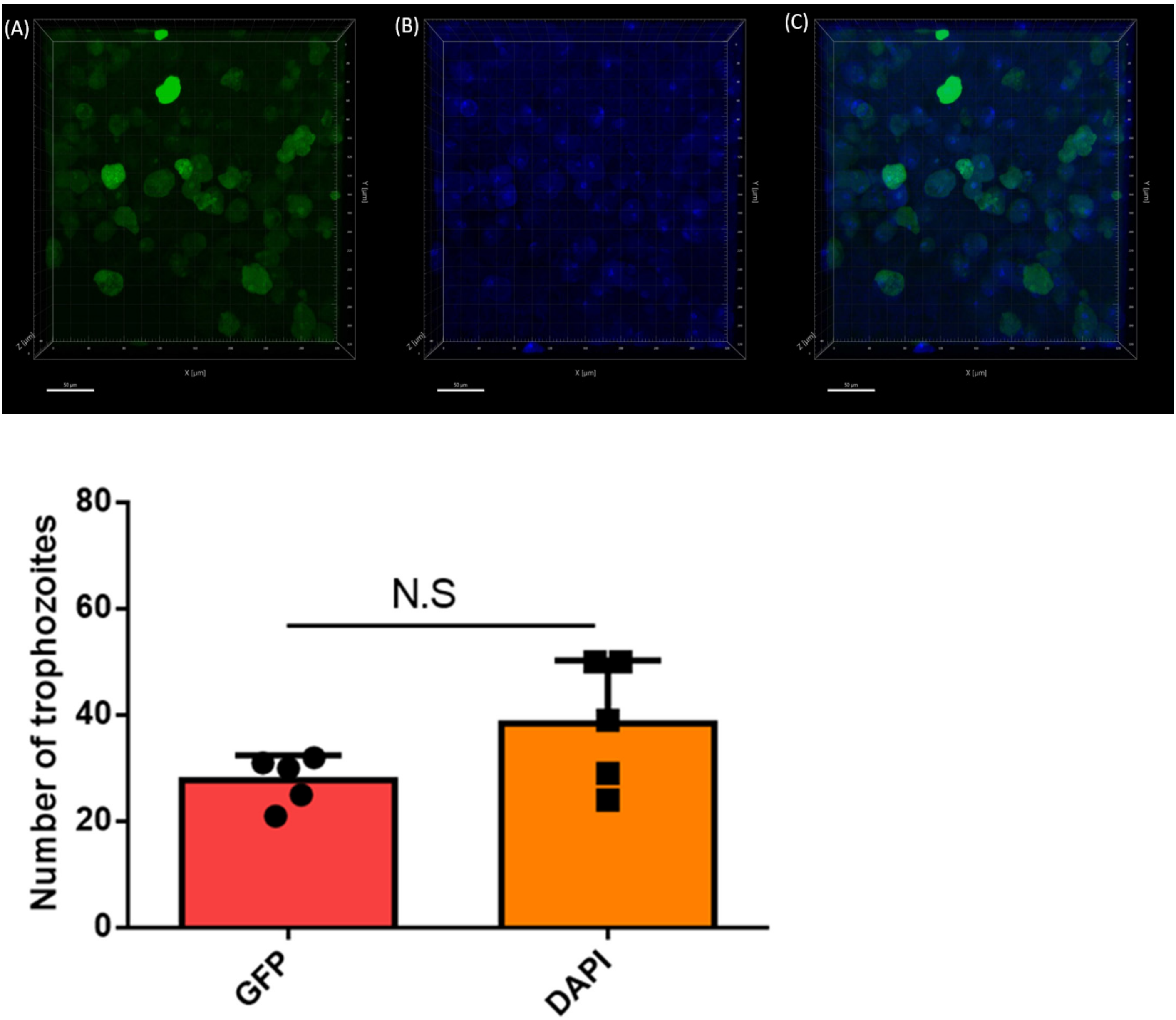

Supplementary Figure 13: Confocal microscopy analysis of trophozoites expressing GFP and trophozoites stained with DAPI exposed to *B. subtilis* biofilm.

Upper panel: Confocal microscopy images (X30) of *B. subtilis* biofilm after 180 minutes of incubation at 37°C with *E. histolytica* trophozoites: (A) Trophozoites expressing GFP. (B) Trophozoites stained with DAPI. (C) Merge pictures.

Lower panel: Quantification of the number of trophozoites present on the biofilm with GFP or DAPI staining, Using ImageJ to count the cells. Statistical analysis was performed using an unpaired T-test P-value is 0.105, data are not significant.

Original gel: Figure 6B

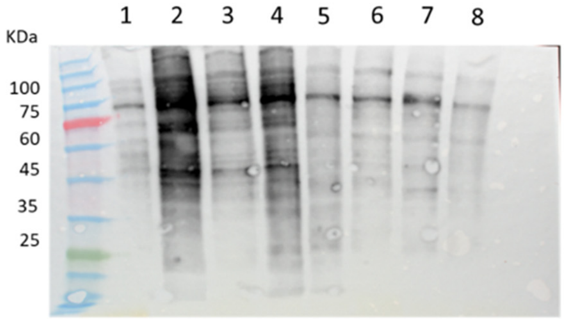

Number	Condition
1	WT
2	WT +H2O2
3	WT + Planktonic
4	WT + Planktonic + H2O2
5	WT + Biofilm RS
6	WT + Biofilm RS +H2O2
7	WT + Biofilm SinR
8	WT + Biofilm SinR +H2O2

Original gel: Supplementary figure 12

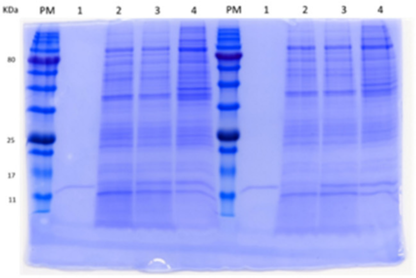

Curli degradation by *E. histolytica* total lysate.

(1) Control Curli
(2) Control trophozoites (WT) lysate.
(3) Curli (2 ug) + WT lysate (20 ug).
(4) Curli (2 ug) + lysate of E64D treated trophozoites (20 ug).

Original gel: Supplementary figure 11

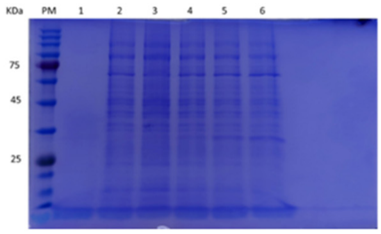

1 : Control TasA
2: control Amoeba
3: Amoeba + 2 ug TasA
4: Amoeba + 5 ug TasA
5: Amoeba + 10 ug TasA
6: Amoeba + 15 ug TasA

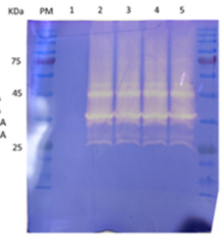

1 : Control TasA
2: control Amoeba
3: Amoeba + 2 ug TasA
4: Amoeba + 5 ug TasA
5: Amoeba + 10 ug TasA

Supplementary Figure 14: Uncropped gels used for the preparation of this manuscript.

Supplementary Tables

Supplementary Table 1: RNAseq data of WT trophozoites vs. WT trophozoites incubated with planktonic *B. subtilis* vs. WT trophozoites incubated with *B. subtilis* biofilms. Results are provided an independent Excel file titled "Supplementary Table 1".

Supplementary Table 2: Gene Ontology IDs used for the annotation of cysteine proteases and Dehydrogenase focused Volcano-plots.

Biological Function	Computed GO Function IDs	Computed GO Functions
Cysteine Proteases	GO:0004197	cysteine-type endopeptidase activity
Cysteine Proteases	GO:0004198	calcium-dependent cysteine-type endopeptidase activity
Cysteine Proteases	GO:0008234	cysteine-type peptidase activity
Dehydrogenase	GO:0000286	alanine dehydrogenase activity
Dehydrogenase	GO:0003854	3-beta-hydroxy-delta5-steroid dehydrogenase activity
Dehydrogenase	GO:0003865	3-oxo-5-alpha-steroid 4-dehydrogenase activity
Dehydrogenase	GO:0003955	NAD(P)H dehydrogenase (quinone) activity
Dehydrogenase	GO:0004022	alcohol dehydrogenase (NAD+) activity
Dehydrogenase	GO:0004029	aldehyde dehydrogenase (NAD+) activity
Dehydrogenase	GO:0004043	L-aminoadipate-semialdehyde dehydrogenase activity
Dehydrogenase	GO:0004152	dihydroorotate dehydrogenase activity
Dehydrogenase	GO:0004303	estradiol 17-beta-dehydrogenase activity
Dehydrogenase	GO:0004352	glutamate dehydrogenase (NAD+) activity
Dehydrogenase	GO:0004471	malate dehydrogenase (decarboxylating) (NAD+) activity
Dehydrogenase	GO:0004617	phosphoglycerate dehydrogenase activity
Dehydrogenase	GO:0008106	alcohol dehydrogenase (NADP+) activity
Dehydrogenase	GO:0008667	2,3-dihydro-2,3-dihydroxybenzoate dehydrogenase activity
Dehydrogenase	GO:0008774	acetaldehyde dehydrogenase (acetylating) activity
Dehydrogenase	GO:0008943	obsolete glyceraldehyde-3-phosphate dehydrogenase activity
Dehydrogenase	GO:0016615	malate dehydrogenase activity
Dehydrogenase	GO:0017113	dihydropyrimidine dehydrogenase (NADP+) activity
Dehydrogenase	GO:0030060	L-malate dehydrogenase activity
Dehydrogenase	GO:0046553	D-malate dehydrogenase (decarboxylating) activity
Dehydrogenase	GO:0047545	2-hydroxyglutarate dehydrogenase activity
Dehydrogenase	GO:0050112	inositol 2-dehydrogenase activity
Dehydrogenase	GO:0050243	pyruvate dehydrogenase (NADP+) activity

Supplementary Table 3: Primers used for Q-PCR

Gene Name	Gene symbol	Oligonucleotide sequence 5’	Oligonucleotide sequence 3’
EhCP4	EHI_168240	CCAGAATCTGTTGATTGG AGA	GCAACCAACAATCTTCCTTC
EhCP5	EHI_050570	CAGAAGGACCAGTTGCT GTT	ATATCCTACAGCGGCAACAC
EhCP6	EHI_151440	TTGCTATTGATGCAGGTC AA	AGATCCATATCCAACAGCACA
Actin	EHI_142730	TTAACTGAAAGAGGATAT GCT	T TCACTGCTTGATGCAGCTTTTT G
